# Salt tolerance in *indica* rice cell cultures depends on a fine tuning of ROS signalling and homeostasis

**DOI:** 10.1371/journal.pone.0213986

**Published:** 2019-04-30

**Authors:** Bushra Ijaz, Elide Formentin, Beatrice Ronci, Vittoria Locato, Elisabetta Barizza, Muhammad Zeeshan Hyder, Fiorella Lo Schiavo, Tayyaba Yasmin

**Affiliations:** 1 Department of Biosciences, COMSATS University Islamabad, Islamabad, Pakistan; 2 Department of Biology, University of Padova, Padova, Italy; 3 Department of Scienze biochimiche e della nutrizione, University Campus Bio-Medico Rome, Italy; National Taiwan University, TAIWAN

## Abstract

Among cereal crops, salinity tolerance is rare and complex. Multiple genes control numerous pathways, which constitute plant’s response to salinity. Cell cultures act as model system and are useful to investigate the salinity response which can possibly mimic a plant’s response to stress. In the present study two indica rice varieties, KS-282 and Super Basmati which exhibited contrasting sodium chloride (NaCl) stress response were used to establish cell cultures. The cell cultures showed a contrasting response to salt stress at 100 mM NaCl. High level of intracellular hydrogen peroxide (H_2_O_2_) and nitric oxide (NO) were observed in sensitive cell culture for prolonged period as compared to the tolerant cells in which an extracellular H_2_O_2_ burst along with controlled intracellular H_2_O_2_ and NO signal was seen. To evaluate the role of NO in inducing cell death under salt stress, cell death percentage (CDP) was measured after 2-4-carboxyphenyl-4,4,5,5-tetramethylimidazoline-1-oxyl-3-oxide (cPTIO) pre-treatment. CDP was reduced significantly in both tolerant and sensitive cell cultures emphasizing NO’s possible role in programmed cell death. Expression analysis of apoplastic NADPH oxidase, i.e. *OsRbohA* and recently characterised OSCA family members i.e. *OsOSCA 1*.*2* and *OsOSCA 3*.*1* was done. Intracellular H_2_O_2_/NO levels displayed an interplay between Ca^2+^ influx and ROS/RNS signal. Detoxifying enzyme (i.e. ascorbate peroxidase and catalase) activity was considerably higher in tolerant KS-282 while the activity of superoxide dismutase was significantly prominent in the sensitive cells triggering greater oxidative damage owing to the prolonged presence of intracellular H_2_O_2_. Salt stress and ROS responsive TFs i.e. *OsSERF1* and *OsDREB2A* were expressed exclusively in the tolerant cells. Similarly, the expression of genes involved in maintaining high [K^+^]/[Na^+^] ratio was considerably higher and earlier in the tolerant variety. Overall, we suggest that a control over ROS production, and an increase in the expression of genes important for potassium homeostasis play a dynamic role in salinity tolerance in rice cell cultures.

## Introduction

Aerobic metabolic processes such as respiration, photosynthesis and photorespiration unavoidably produce reactive oxygen species (ROS) in the mitochondria, chloroplast, and peroxisomes respectively [1–2]. These ROS are produced in a controlled amount under optimal conditions. However, under abiotic stress their level increases dramatically. Overproduction of ROS caused by abiotic stress in plants highly damages proteins, lipids, and nucleic acids leading to cell injury and death [2]. ROS are also generated across the plasma membrane and apoplastic region [1–3]. Under abiotic stress these apoplastic ROS might also act as signal molecules for the activation of stress responsive pathways [4].

ROS induced by salt stress have lately been gaining more attention as second messengers [5–6]. Salt-induced ROS are generally represented by H_2_O_2_ [7], mainly produced at the apoplast by calcium or phosphorylation derived activation of plant NADPH oxidases (NOXs) also known as respiratory burst oxidase homologs (RBOHs) [8]. This NOX generates a ROS signal which moves to the cytoplasm via regulated aquaporin [9], and together with intracellular ROS alters the redox status of key regulatory proteins such as transcription factors (TFs) [10]. This ROS signal activates numerous signaling transduction pathways to mediate multiple biological processes, including abiotic stress response and adaptation [11–12]. The elevated levels of ROS during the early phase of stress may act as a vital signal but the regulatory components of ROS mediated stress response are unknown. Yet, a signal transduction pathway has been proposed in which a mitogen-activated protein kinase (MAPK) cascade and downstream TFs are the key regulators of ROS signaling [13,7].

Rice is a highly salt sensitive crop, and its growth is severely affected when the plant is exposed to saline stress [14]. Owing to its large genetic variability, rice species show different degrees of salt sensitivity [15]. Seedling and reproductive phases of growth are the most sensitive stages under salinity [16]. Salt exerts its toxicity by inducing osmotic, ionic and oxidative stress [17–18]. Excessive Na^+^ entry depolarizes the plasma membrane, increases Ca^2+^ influx through unknown channels causing a change in Ca^2+^ signature of the cell activating a signaling cascade. In response, the cells try to minimize Na^+^ uptake, increase Na^+^ sequestration and extrusion and restore K^+^ levels by the up-regulation of Na^+^/K^+^ symporter and antiporter [19,20]. An enhanced detoxification of ROS is also activated at the same time to reduce the oxidative damage [21–23].

In rice, NOX-dependent H_2_O_2_ production emerges within few minutes of salt stress [24] and generates the earliest defence response at the molecular level. Salt and H_2_O_2_ responsive ethylene response factor TFs, *SERF1* and Dehydration-Responsive Element Binding 2a (*DREB2a*) have a critical role in regulating H_2_O_2_ mediated molecular signalling cascade during the early response to salt stress in rice and Arabidopsis respectively [25,26]. *SERF1* is essential for the proliferation of the first ROS signal in rice roots [27] while an early and enhanced expression of DREB2a evidently controls stress responses through abscisic acid “ABA” independent signaling, improving dehydration and tolerance to salt stress in rice plant [28].

An increased understanding of ROS signals generation and propagation is required to gain further insight into the regulatory mechanism underlying responses and adaptation to salt stress in cereal crops.

The aim of the study is four-fold. Firstly, we developed cell cultures from the mature seeds of two indica rice varieties to provide a simplified model that can accurately mimic the response of rice plant under salt stress. Secondly, it attempts to quantify the amounts of redox signalling molecules H_2_O_2_ & NO produced extracellularly as well as intracellularly in sensitive/tolerant cells/varieties. Thirdly, it describes the expression pattern of salt-responsive apoplastic NOX subunit *OsRbohA* and TFs *OsSERF1* and *OsDREB2A* and their correlation with the ROS signature of the cell. Fourthly, we tried to explore the role of recently characterised OSCA1 family members important for initial Ca^2+^ influx (*OsOSCA 1*.*2* and *OsOSCA 3*.*1*) and genes important for Na^+^ and K^+^ homeostasis (i.e. *OsNHX1*, *OsSOS1*, *OsTpka*, *OsHAK5* and *OsKAT*) to understand their role for survival under salt stress.

## Materials and methods

### Plant material and salt treatment

Experiments were conducted with non-embryogenic cell cultures of two rice (*Oryza sativa* L.) varieties, KS-282 and Super Basmati. Briefly, mature seeds were dehusked and surface sterilized under the laminar hood with 70% ethanol for 1 minute followed by 15 min in 3.5% sodium hypochlorite and tween-20 on a gyratory shaker, followed by 5 min with 3.5% bleach followed by five washes with Mille-Q water, 2 min each.

After surface sterilization, seeds were sowed in solid N6 medium (3.96 g L^−1^ Chu(N6) Medium Salt Mixture, 30 g L^−1^ sucrose, 2 mg L^−1^ 2,4-dichlorophenoxyacetic acid (2,4-D) and 8 g L^−1^ plant ager; pH 5.8) under dark conditions and moved to new agar plates fortnightly. After few months, the callus was friable, and ready to be transferred in the liquid medium. The resulting Cell cultures were grown at 26°C in dark on a rotatory shaker at speed of 96rpm in liquid N6 medium (3.96 g L^−1^ Chu(N6) Medium Salt Mixture, 30 g L^−1^ sucrose and 2 mg L^−1^ 2,4-dichlorophenoxyacetic acid (2,4-D) pH 5.8). Every ten days they were filtered to eliminate bigger clumps until quite homogeneous Cell cultures were obtained. For the next sub culturing, 2 mL of packed cell volume of cells was transferred into 50 mL fresh medium every 10 days. Cells at day 3 after subculture were treated with 75, 100 and 150 mM NaCl and used for the next experiments. To determine the growth capabilities of the two cell cultures under salt stress, cells were filtered and the fresh and the dry weight were measured.

### Cell viability assay under control and stress condition

Cell death was evaluated by a spectrophotometric assay of Evans blue stain retained by cells according to Gaff and Okong’o-Ogola [29] with minor modifications. Briefly, one mL of cell cultures was sampled from the cultures at desired intervals. Evans blue dye solution was added to the cell cultures to a final concentration of 0.05% and incubated for 30 min at room temperature, followed by rigorous washing with water until the disappearance of colour. The 50% methanol containing 1% (w/v) sodium dodecyl sulfate (SDS) was added to the washed cells and then incubated at 56°C for 30 min. The percentage of dead cells was determined spectrophotometrically by measuring/at OD 600 nm. The boiled cells (100% dead) were used for comparison.

### ROS and RNS assay

#### Determination of extracellular hydrogen peroxide

Extracellular H_2_O_2_ secreted in the medium by the cultured cells was measured as described by Bellincampi (2000) [30]. Briefly, 500ul of the culture medium from control and treatment cell cultures was added (1:1) assay reagent (500 μM (NH4)2Fe(SO4)2·6H2O, 50 mM H_2_SO_4_, 200 μM xylenol orange, and 200 mM sorbitol) and kept in dark for 45 min. The H_2_O_2_ dependent oxidation of Fe^2+^ to Fe^3+^ was determined by measuring the absorbance Fe^3+^-xylenol orange (FOX) complex at 560nm. A calibration curve obtained by measuring the absorbance of FOX complex of H_2_O_2_ standards allowed the conversion of the absorbance values into concentration estimates. All reactions were carried out at least in duplicate with four replicates to check their reproducibility.

#### Determination of intracellular hydrogen peroxide

Intracellular H_2_O_2_ production was measured using dihydrorhodamine123 (Sigma-Aldrich, Germany) as a probe. 1ml of cell cultures was incubated with 20 μM DHR123 for 15 min in a rotating shaker, and then washed thrice with 1 mL of fresh N6 medium. The cells were analysed under a fluorescence microscope (DM5000, Leica Microsystems, Germany) with a I3 filter. Relative fluorescence was determined using the ImageJ software.

### Enzyme activity assay

The activity of ROS scavenging enzyme i.e. ascorbate peroxidase (APX) (total (tAPX) and cytosolic(cAPX)), catalase (CAT), glutathione reductase (GR), monodehydroascorbate reductase (MDHAR), dehydroascorbate reductase (DHAR) and superoxide dismutase (SOD) was measured. 0.5 g of cells were harvested at 30 min and 24 h and ground using a sterilized pistil and mortar with liquid nitrogen. Powdered cells were homogenized at 4°C in an extraction buffer (50 mM Tris-HCl pH 7.5, 0.05% cysteine, 0.1% bovine serum albumin). Homogenates were centrifuged at 14000 g for 15 min at 4°C to obtain supernatant which was used for enzyme activity. Protein contents were determined according to Bradford (1976) [31] using bovine serum albumin (BSA) as a standard. The activity of SOD was measured according to Beauchamp and Fridovich (1971) [32].

### Determination of intracellular Nitric Oxide

Intracellular NO was detected with the fluorescent dye 4-Amino-5-Methylamino-2',7'-Difluorofluorescein Diacetate (DAF-FM-DA; Alexis Biochemical). One ml of cells was incubated with 0.5 mM DAF-FM-DA for 15 min in a rotating shaker, and then cells were washed three times with 1 mL of fresh N6 medium. Fluorescence was estimated using a fluorescence microscope (DM5000, Leica Microsystems, Germany) with a green florescent protein (GFP) filter. Relative fluorescence was determined using the ImageJ software.

### cPTIO pre-treatment and CDP analysis

50 **μ**M cPTIO was added directly to the cells cultures and incubated for 15 min on a rotatory shaker. Cell cultures were then exposed to 100 mM NaCl and cell death was evaluated by a spectrophotometric assay of Evans blue stain retained by cells according to Gaff and Okong’o-Ogola (1971) [29] with minor modifications.

### Total RNA isolation and first strand cDNA synthesis

Total RNA was extracted from both cell cultures using the RNeasy Plant Mini Kit followed by in-column DNase treatment (Qiagen, Hilden, Germany). RNA concentrations were measured using a Nano drop ND-1000 spectrophotometer (Nano drop Technologies, Rockland, DE, USA) and 5 μg of RNA was treated with RQ1 DNase (Promega) according to the manufacturer’s protocols. RNA was precipitated with 2.2 μl of CH_3_COONa 3M pH 5.2 and 60.5 μl of ethanol and incubated for 30 min at -20°C. After centrifugation the pellet was washed with 70% ethanol, air-dried, re-suspended in 5 μl of water and used for first-strand cDNA synthesis using the Superscript II Reverse Transcriptase (Thermo Fisher) according to user instructions.

### Gene expression profile analysis

RT-qPCRs were performed using GoTaq qPCR master mix (Promega, United States) on the QuantStudio 12K Flex real time PCR system with standard protocol. Ubiquitin (Os05g0160200) was used as reference gene. Primers are listed in supporting information, S1 Table.

### Ion analysis

Cells from control and treatment groups were harvested at 12, 24 and 72 h. Cells were filtered and oven dried at 40°C for three days The dry weight was recorded and then the cells were ground to a fine powder which was afterwards dissolved in 5ml of 68% HNO_3_ (Sigma Aldrich, Germany). Standard calibration curve was obtained for Na^+^ and K^+^ using 1000ppm Stock solutions (Fisher Scientific, USA). The samples were analysed using Atomic absorption spectrophotometer (Perkin Elmer Model 303). The concentration of Na^+^ and K^+^ (μg/g) was determined by comparing to the known concentrations of cation standards.

### Statistical analysis

The data was analysed by two-way ANOVA *(p<0*.*05)* followed by Bonferroni post hoc test. The comparative C_T_ method was used to analyze the gene relative expression (ΔΔC_T_ method, [33]).

## Results

### Salt sensitivity/tolerance of developed rice cell cultures

Cell cultures from two rice varieties were successfully developed and stabilized to study the mechanisms of salt tolerance at the cellular level. The two cell cultures were exposed to three salt concentrations (75, 100, 150 mM), and growth parameters along with measurements of cell death were evaluated.

**Fig 1:**
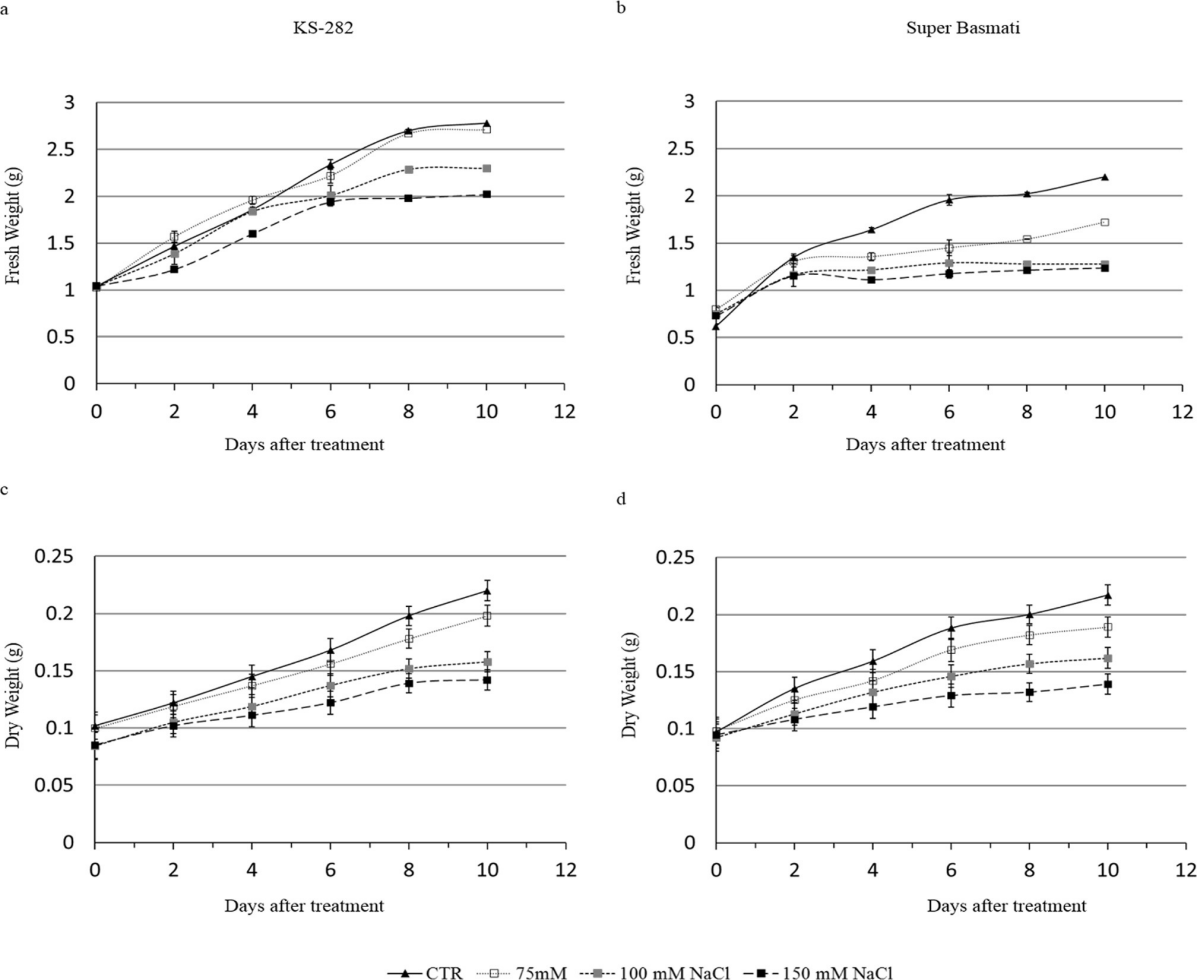
Fresh weight (g) and Dry weight (g) of cultured cells at different time points after NaCl treatment. Three days after sub culture the cells of KS-282 (a,c) and Super Basmati (b,d) were treated with different concentrations of NaCl. Closed triangles–straight line, Control; open squares–dotted line, 75 mM NaCl; grey squares–small-dashed line, 100 mM NaCl; closed squares–dashed line, 150 mM NaCl. The fresh weight was taken immediately after filtering the cell cultures. The dry weight was measured after drying the cells for 3 days at 40°C. Values represent the mean ± SE (p<0.05) of three independent experiments performed in triplicate. The differences reported between the control and treated groups were statistically significant according to the two-way ANOVA followed by Bonferroni post hoc test.

By comparing the growth curves of the two cell cultures, differences in salt sensitivity were observed (Fig 1). A difference in the fresh weight of the two varieties was observed under control conditions. KS-282 showed a higher growth rate (Fig 1A) than Super Basmati (Fig 1B) at day 10. There was significant effect of salinity levels on the average calli fresh weights of both varieties. The fresh weight of Super Basmati reduced significantly as the salinity level increased from 75 to 100 and 150 mM. No significant difference in the growth rate of KS-282 cell cultures under control and at 75mM NaCl stress was observed, however the rate of cell division was significantly affected at 100 and 150 mM NaCl starting from day 6. The sensitive Super Basmati responded to all stress levels from day 4 showing severely retarded growth under saline condition. A similar drop in the dry weight of cell cultures was observed in both cell cultures under different salt concentrations (Fig 1C and 1D).

**Fig 2:**
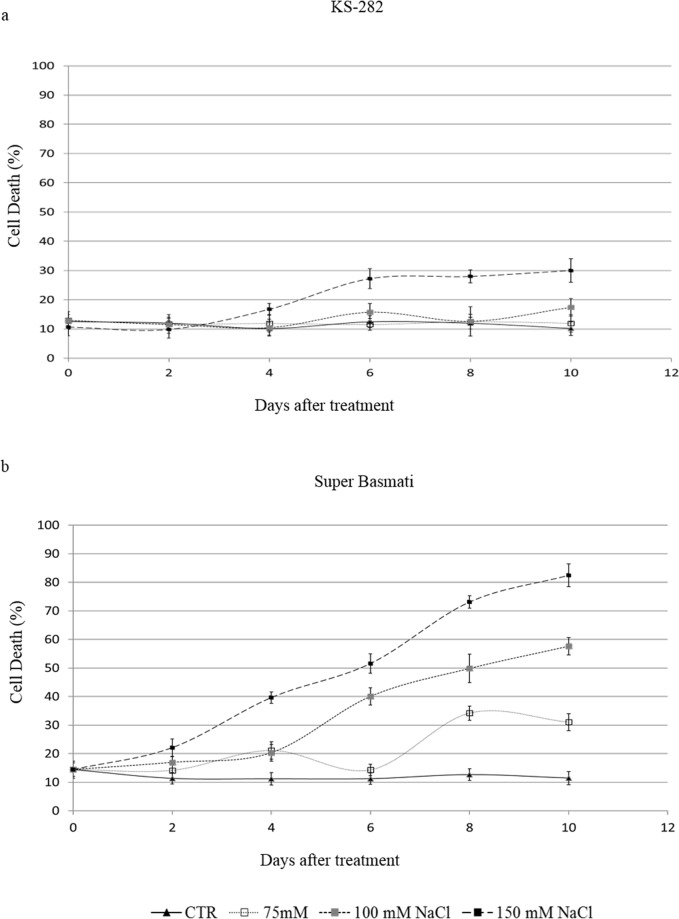
Survival curves of KS-282 and Super Basmati cultured cells in presence and absence of NaCl. Three days after sub culture the cells of KS-282 (a) and Super Basmati (b) were treated with different concentrations of NaCl. Closed triangles–straight line, Control; open squares–dotted line, 75 mM NaCl; grey squares–small-dashed line, 100 mM NaCl; closed squares–dashed line, 150 mM Cell viability and cell death percentage was measured using Evans blue staining. Values represent the mean ± SE (p<0.05) of three independent experiments performed in triplicate. The differences reported between the control and treated groups were statistically significant according to the two-way ANOVA followed by Bonferroni post hoc test.

The measurement of cell death percentage (CDP) at different time points during subculture cycles showed that in Super Basmati CDP reached 40% and 52% after day 6 of salt treatment, depending upon the salt concentration (100 and 150 mM NaCl, respectively; Fig 2B). KS-282 cells, on the other hand, showed a slow increase in the CDP (27% at 150mM NaCl) at day 6 characterizing it as the tolerant cell culture (Fig 2A). No significant differences in cell viability of KS-282 cell cultures were observed in the presence of 75, 100 mM NaCl.

### Extracellular hydrogen peroxide

**Fig 3:**
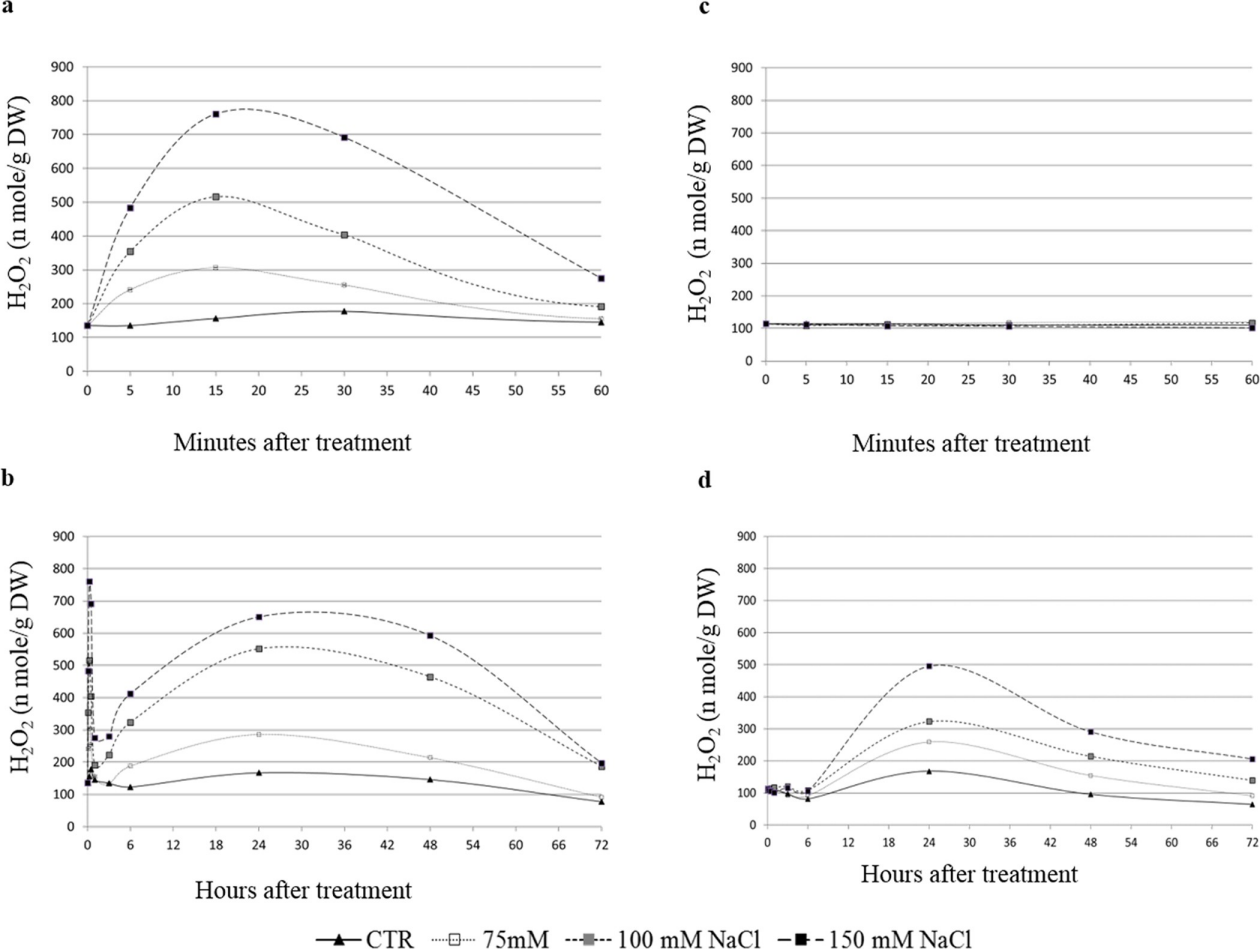
Extracellular H_2_O_2_ produced by rice cell cultures. H_2_O_2_ released in the media by the cell cultures of KS-282 (a and b) and Super Basmati (c) measured at different time points after salt treatment. Closed triangles–straight line, Control; open squares–dotted line, 75 mM NaCl; grey squares–small-dashed line, 100 mM NaCl; closed squares–dashed line, 150 mM NaCl. DW, Dry weight. Values represent the mean ± SE (p<0.05) of at least three independent experiments. The differences reported between the control and treated groups were statistically significant according to the two-way ANOVA followed by Bonferroni post hoc test.

The H_2_O_2_ produced apoplastically was measured to observe the level of H_2_O_2_ production by the cell cultures under control and NaCl stress. Around 100 nmol/g dry weight of H_2_O_2_ was measured as basic level produced in the culture medium of untreated cells. In case of tolerant KS 282 cells, we were able to see a burst of H_2_O_2_ with a first peak at 15 min (Fig 3A) and another at 24 h (Fig 3B) with a decreasing amplitude after 24 h for all three concentrations (75, 100 and 150 mM NaCl; p<0.05, two-way ANOVA, between control and treated cells) of salt. However, the sensitive cell cultures produced a single peak of H_2_O_2_ at 24 h (Fig 3C). H_2_O_2_ was not detected during the first 6 h of salt treatment in culture medium of sensitive Super Basmati for all salt concentrations. Notably, H_2_O_2_ production occurred in a dose-dependent manner in both cell cultures.

### Intracellular hydrogen peroxide

**Fig 4:**
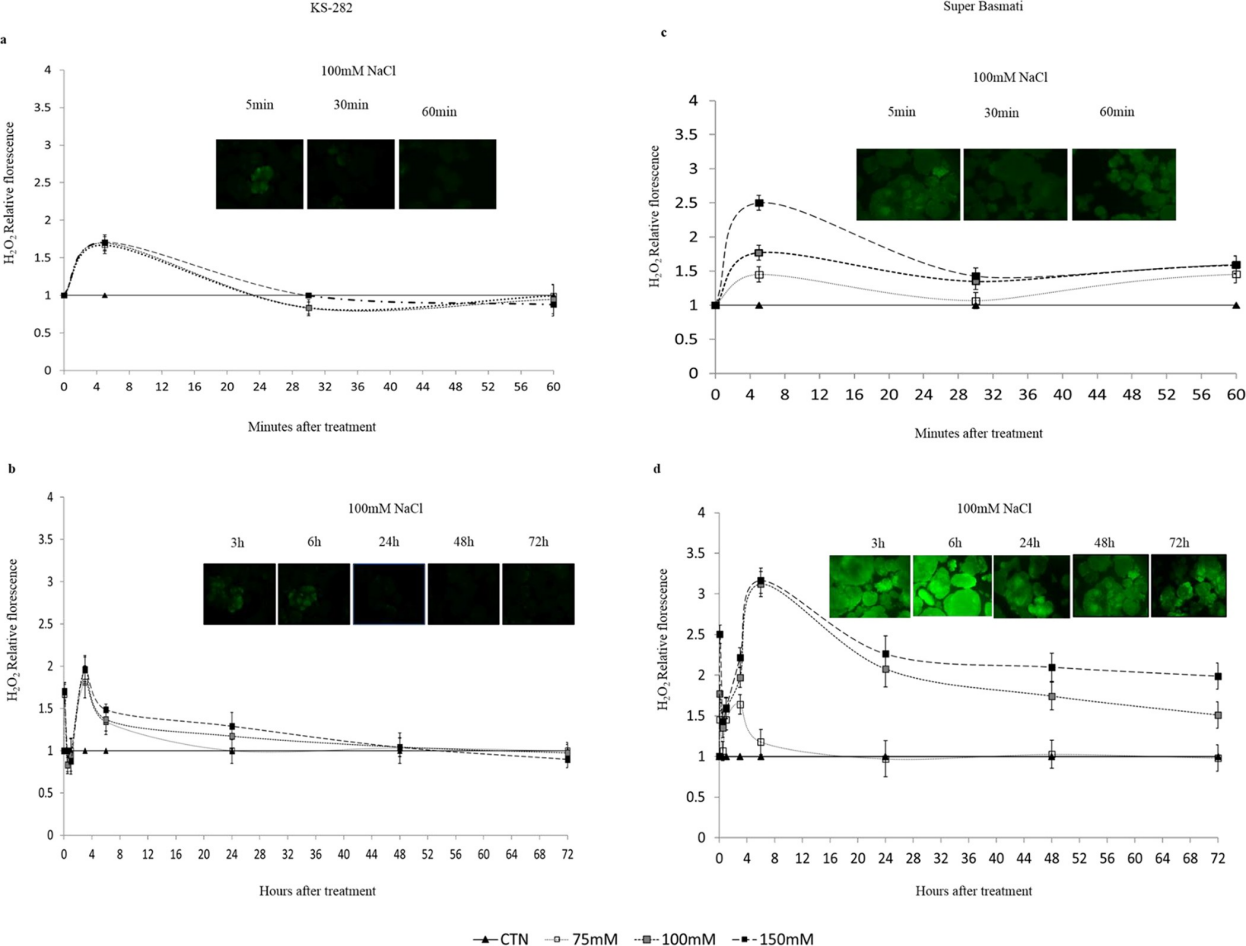
Intracellular H_2_O_2_ produced by cell cultures. H_2_O_2_ produced intracellularly by the cell cultures of KS-282 (a and b) and Super Basmati (c and d) as measured at different time points after salt treatment. Closed triangles–straight line, Control; open squares–dotted line, 75 mM NaCl; grey squares–small-dashed line, 100 mM NaCl; closed squares–dashed line, 150 mM NaCl. DW, Dry weight. The measurement was done using DHR-123 as florescent probe. Values were normalized against the levels of control cells, which are given a value of 1 and therefore have no SD. Values represent the mean ± SE (p<0.05) of at least two independent experiments. The differences reported between the control and treated groups were statistically significant according to the two-way ANOVA followed by Bonferroni post hoc test.

Since the level of H_2_O_2_ decides the fate of cell under salt stress [4], the intracellular level of H_2_O_2_ was measured. The cells of tolerant and the sensitive varieties showed two peaks of H_2_O_2_ inside the cells (Fig 4). One small peak was observed at 5 min with a decrease in H_2_O_2_ level within 30 min of salt treatment. In tolerant KS-282 cell cultures the level of first peak was lower (Fig 4A) under all salt concentrations as compared to the sensitive variety. The second peak was observed at 3 h (Fig 4B) of salt treatment with a low amplitude in a dose dependent manner which reduced progressively and reached the basal level at 24 h. On the other hand, in the more sensitive cell culture, salt treatment induced a second delayed sustainable peak with maximum amplitude at 6 h (Fig 4D). The level of H_2_O_2_ remained high above the optimal levels at 72 h. The production of intracellular H_2_O_2_ again occurred in a dose-dependent manner in both the cultivars.

### Enzyme activity assays

**Fig 5:**
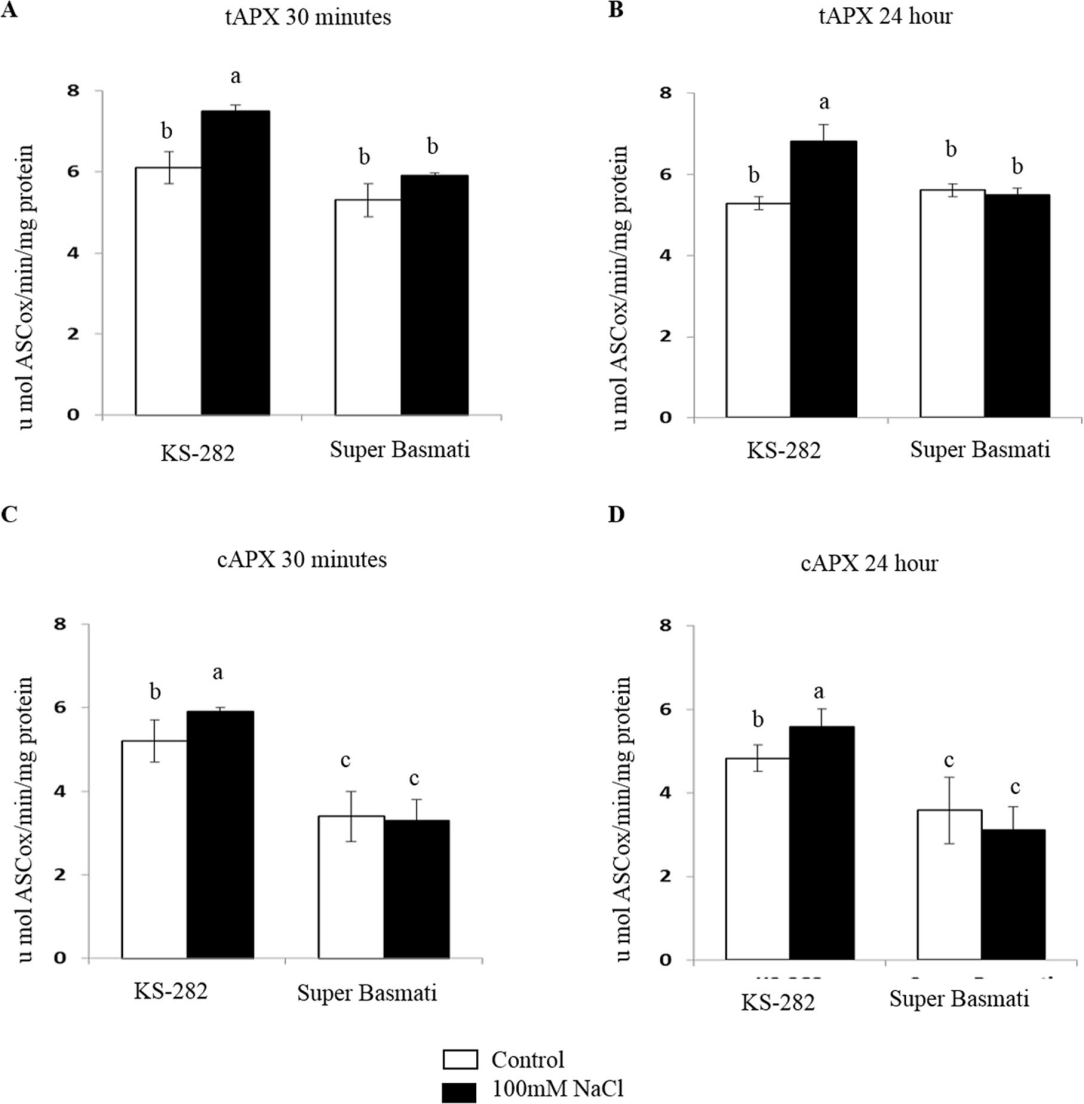
Antioxidant enzyme activities in control and treated cells. Activity of total (a and b) and cytosolic (c and d) APXs after 30 min (left) or 24 h (right) of treatment with 100 mM NaCl. Values represent the mean ± SE of three independent experiments. Different letters indicate significantly different activities according to ANOVA (p<0.05).

**Fig 6:**
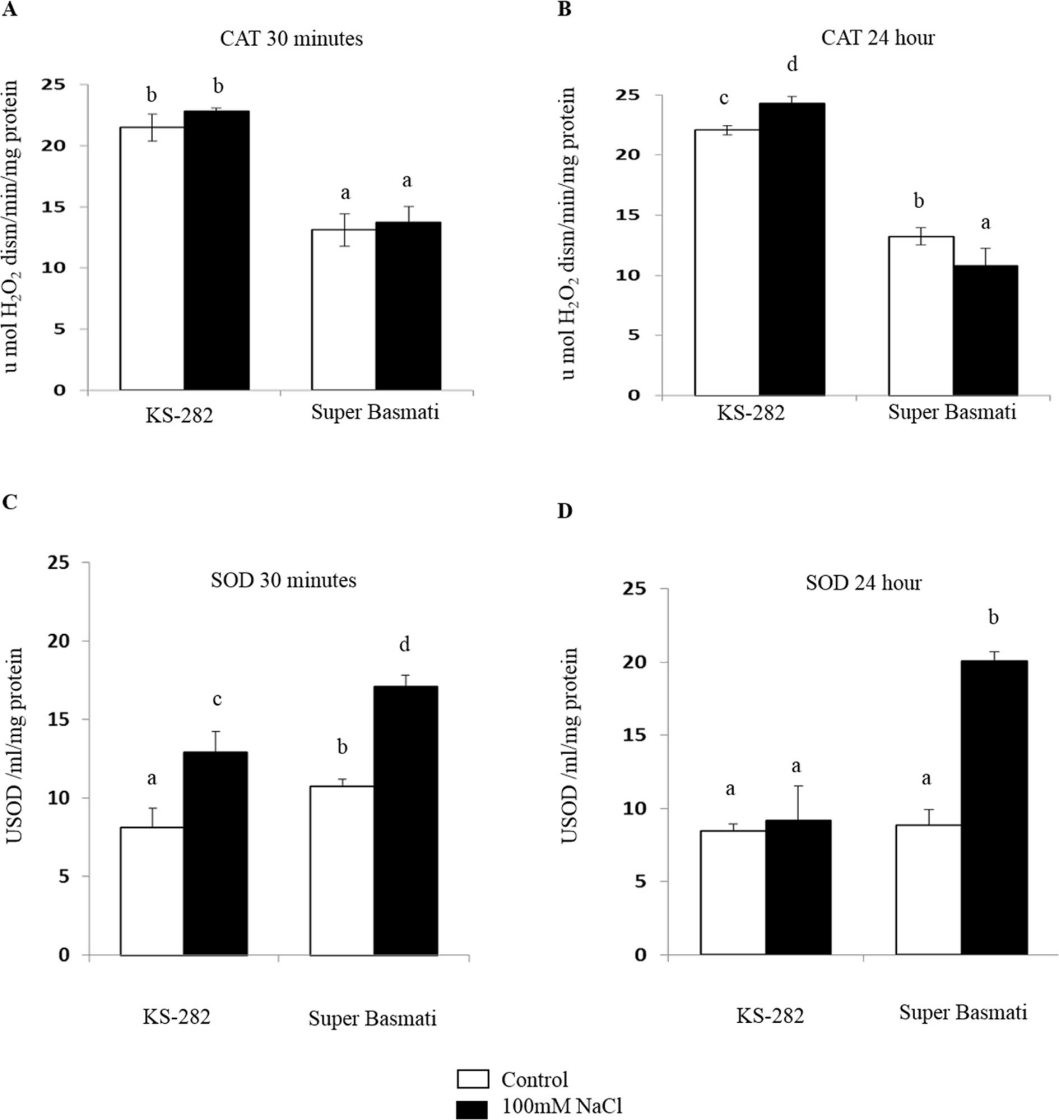
Antioxidant enzyme activities in control and treated cells. Activity of catalases (CAT, a and b) and super oxide dismutase (SOD, c and d) after 30 min (left) or 24 h (right) of treatment with 100 mM NaCl. Values represent the mean ± SE of three independent experiments. Different letters indicate significantly different activities according to ANOVA (p<0.05).

The activity of tAPX, cAPX and CAT was relatively high in the cells of tolerant KS-282 as compared to Super Basmati (Fig 5A–5D and Fig 6A and 6B) while the activity of SOD was comparatively low in the tolerant KS-282 cells as compared to the sensitive cells (control and treated respectively) (Fig 6C and 6D). A significant increase in the activity of tAPX and cAPX was noticed at 30 min and 24 h in NaCl treated cells of tolerant KS-282 while no change was observed in its activity in Super Basmati cell cultures (Fig 5A–5D) exhibiting its low ROS scavenging ability via APX relative to the tolerant KS-282 cells. Similarly, CAT activity remained low in NaCl treated cells of Super Basmati at both time points (13.74± 1.28 and 10.77±1.48 μmol H_2_O_2_ dism/min/mg protein respectively) than the tolerant KS-282 cells. In KS-282 a significant increase was observed at 24 h (24.31± 0.57 μmol H_2_O_2_ dism/min/mg protein) (Fig 6B) of sat treatment. No change in the activity of MDHAR, DHAR and GR was observed at 30 min and 24 h in both KS-282 and Super Basmati control and NaCl treated cells (Supporting Information, S3 Fig and S4 Fig). The activity of SOD in control cells of tolerant and sensitive cell cultures (Fig 6C and 6D) was almost equal (8.28 ± 0.47and 8.97± 1.07-unit SOD/ml/mg protein respectively) while under saline conditions the activity of SOD increased and was higher in sensitive cell culture at 30 min (17.11± 1.29 unit SOD/ml/mg protein) as compared to the tolerant KS-282 cells (12.92 ± 0.72 unit SOD/ml/mg protein). SOD activity was reduced in the tolerant KS-282 cells at 24 h to optimal level (9.21± 0.63unit SOD/ml/mg protein) while it increased to 20.04 ± 2.30-unit SOD/ml/mg protein in Super Basmati sensitive cell culture (Fig 6C and 6D) exhibiting a steady increase in H_2_O_2_ level.

### Intracellular nitric oxide levels

**Fig 7:**
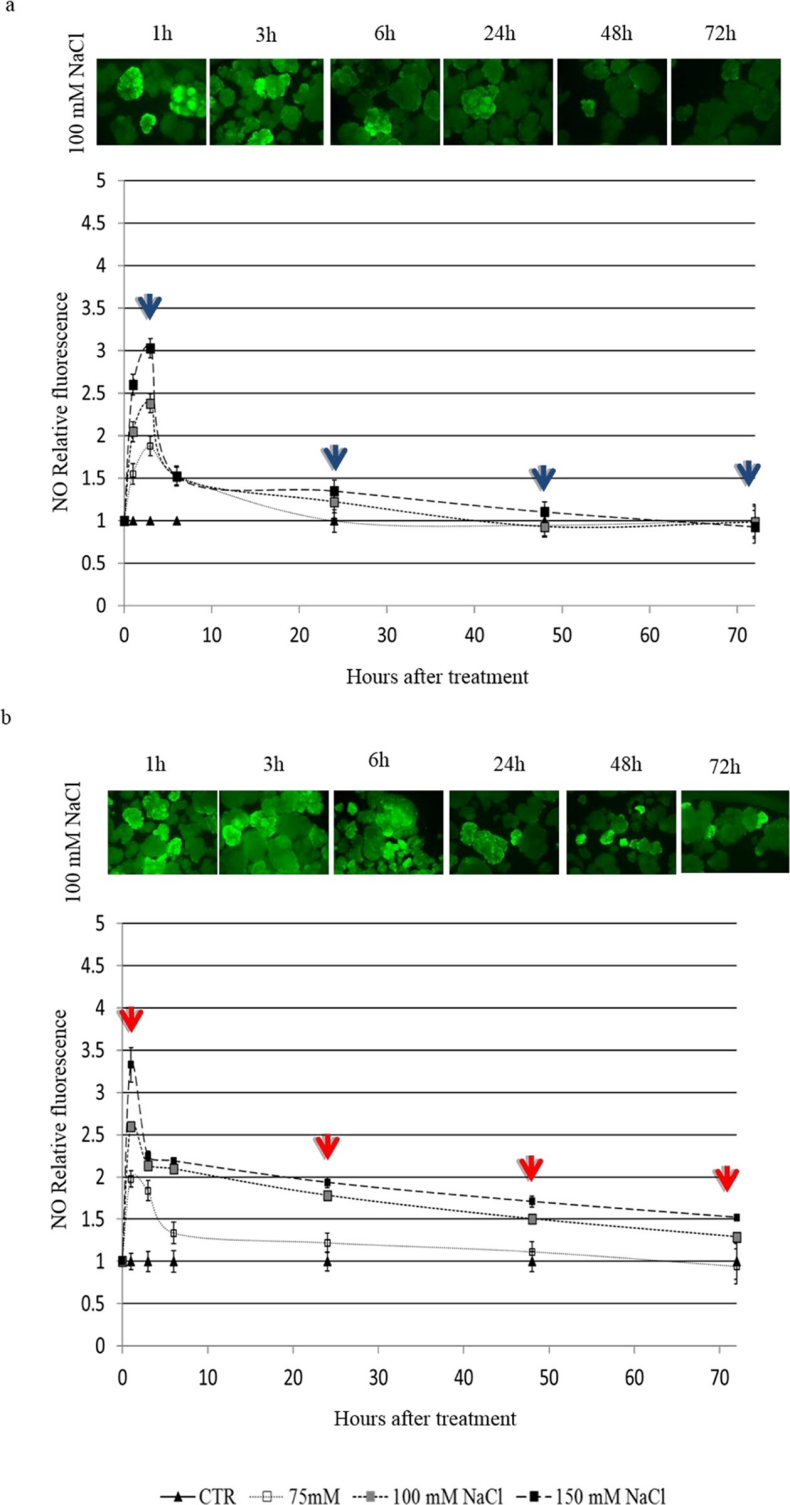
NO produced by rice suspension cell cultures. Relative amount of NO measured by DAF-FM-DA in cells of KS-282 (a) and Super Basmati (b) at different time points after salt treatment. Closed triangles–straight line, Control; open squares–dotted line, 75 mM NaCl; grey squares–small-dashed line, 100 mM NaCl; closed squares–dashed line, 150 mM NaCl. Values were normalized against the levels of control cells, which are given a value of 1 and therefore have no SE. Values represent the mean ± SE of at least two independent experiments. The differences between the control and treatment groups were reported were statistically significant according to the two-way ANOVA at p<0.05 (n>30).

In response to salt stress, NO production was observed in both cell cultures. In case of sensitive Super Basmati cells an initial increase in NO was detected with all three salt concentrations (Fig 7B). This high level dropped after 6 h with 75mM NaCl while with 100 and 150 mM NaCl level of NO remained nearly steady up to 72 h. On the other hand, in KS-282 cells only an early narrow peak of NO (at 3 h) was detected in the presence of all three NaCl concentrations followed sharp reduction in NO level (Fig 7A). The differences reported were statistically significant according to the two-way ANOVA at p<0.05 (n>30).

### CDP measurement after cPTIO pre-treatment

**Fig 8:**
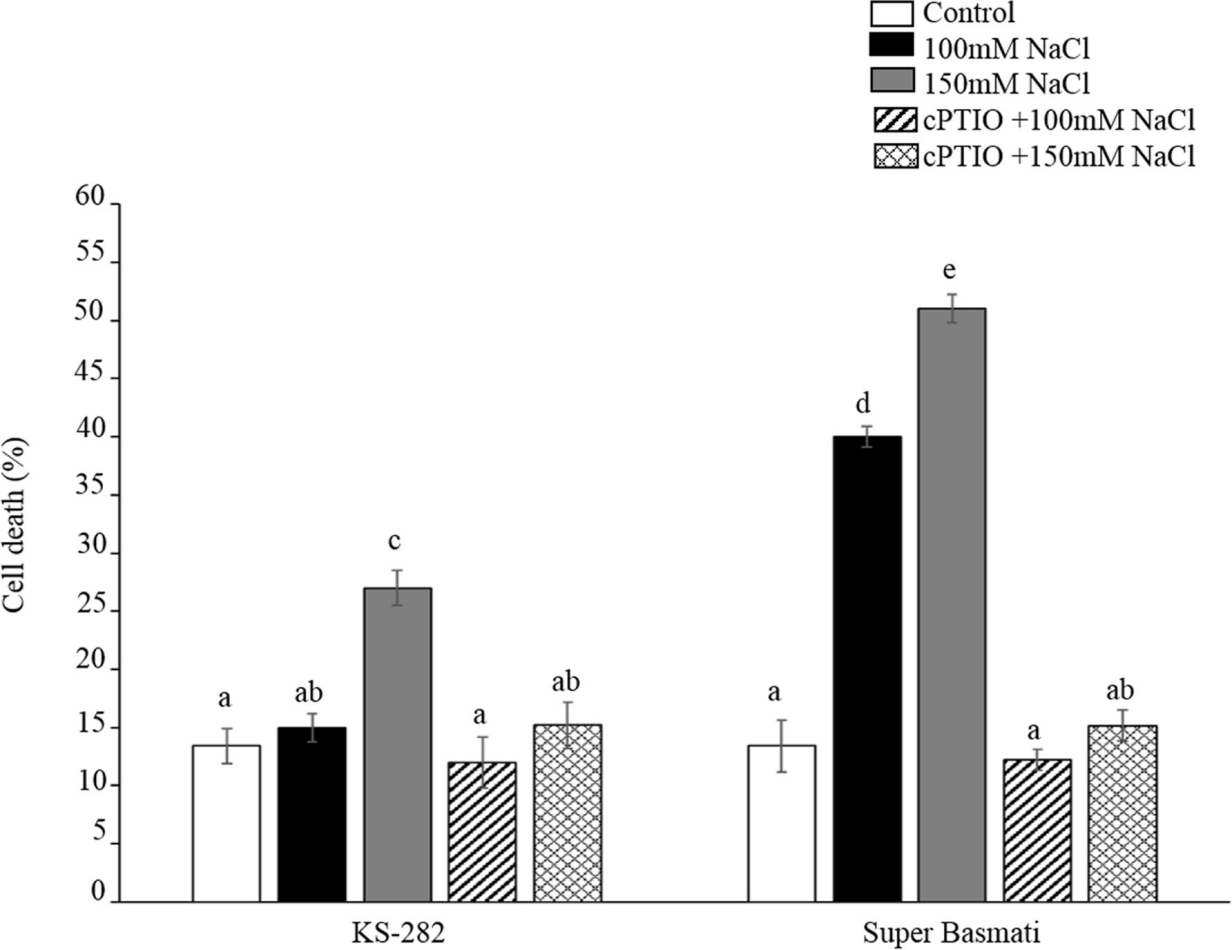
The effect of cPTIO pre-treatment on cell death rate. Cell death rate as measured in tolerant (a) and sensitive (b) cell cultures after 50uM cPTIO pre-treatment and 100 and 150mM NaCl stress. In the pre-treated tolerant KS282 cell cultures the CDP decreased to 23 and 55% at 100 and 150mM NaCl respectively on D6 while in the sensitive Super Basmati cells exposed to 100 mM NaCl, on D6 CDP decreased to 68% as compared to the non-pre-treated cells while under 150mM NaCl the percent decrease in CDP was 71%. The cell viability and cell death percentage was measured using Evans blue staining according to Gaff and Okong’o-Ogola (1971). Values represent the mean ± SE (p<0.05) of three independent experiments performed in triplicate. Different letters indicate statistically different expression levels according to two-way ANOVA (p<0.05).

CDP was measured in cell cultures of both tolerant and sensitive varieties pre-treated with 50uM cPTIO and then exposed to 100mM and 150mM NaCl. cPTIO is a NO radical scavenger and can possibly predict the role of NO in programmed cell death. Viability assay demonstrated a significant reduction in CDP in both tolerant and sensitive cell cultures under both salt concentrations. In sensitive Super Basmati cells exposed to 100 mM NaCl, on day 6 CDP reduced to 68% as compared to the non-pre-treated cells while under 150mM NaCl the percentage reduction in CDP was 71% on day 6 as compared to the non-pre-treated cells at day 6 of salt treatment (Fig 8B). In the tolerant KS-282 pre-treated cells CDP reduced to 23 and 55% at 100 and 150mM NaCl respectively on day 6 (Fig 8B). This marked reduction in CDP demonstrate a possible role of NO radicals in inducing programmed cell death in cell cultures.

### Genes involved in ROS signalling

In our study an extracellular H_2_O_2_ burst was observed early in the tolerant line on salt treatment, whereas a high level of H_2_O_2_ combined with sustained levels of intracellular NO was detected in the sensitive cells. These two responses to salt stress may induce signals that lead to different fates, i.e., the induction of resistant mechanisms in KS-282 cells and the process of programmed cell death (PCD) in Super Basmati cells.

**Fig 9:**
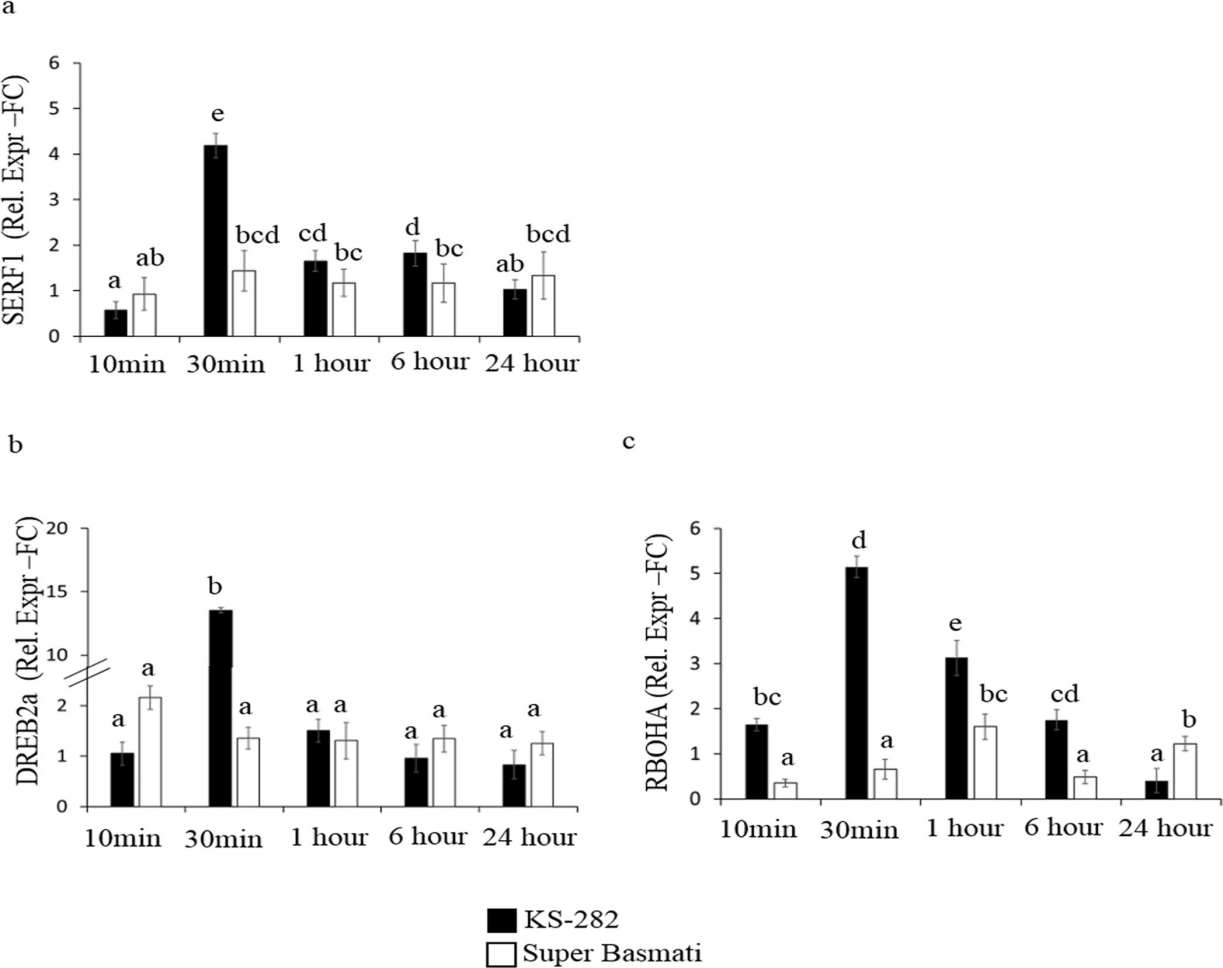
Effect of NaCl on the expression of genes important for signalling response. Relative expression (Fold Change) of (a) *OsSERF1* gene (b) *OsDREB2A* and (c) *OsRboha* gene at different time points after treatment with 100 mM NaCl. Values represent the mean ± confidence interval of three independent experiments in duplicate. Different letters indicate statistically different expression levels according to one-way ANOVA (p<0.05).

To investigate this hypothesis in more detail, the expression of genes linked to ROS signalling pathway was investigated before (Supporting Information, S1C–S1E Fig) and after salt treatment. First, the expression of *Salt-Responsive ERF1*(*SERF1*), the rice TF gene known for being involved in H_2_O_2_ dependent salt stress signalling [25] and its possible role in salt stress adaptation, was analysed. In Super Basmati cells, there was a small peak of expression at 30 min of exposure to salt stress, whereas *OsSERF1* expression in KS-282 cells was significantly high at 30 min of stress (Fig 9A). Another TF, Dehydration Response Element Binding 2A (*OsDREB2A*), activated by ROS and SERF1 and known to improve dehydration and salt stress tolerance in rice [28] was analyzed. *OsDREB2A* was found to be not responsive in sensitive Super Basmati cell cultures while by contrast, cell cultures of tolerant KS-282 showed enhanced expression of *OsDREB2A* at 30 min of salt treatment (Fig 9B).

*OsRbohA* gene expression was analysed in both cell cultures. *OsRbohA* belongs to the respiratory burst oxidase homologues (RBOH; [34]) gene family playing a main role in apoplastic ROS production in plants [12]. In our system, *OsRbohA* was significantly upregulated in KS-282 at 30 min of salt treatment (Fig 9A), whereas in Super Basmati expression of this gene was low event at 24 h (Fig 9C).

### Mechano-sensitive Ca^2+^ channels mediated by oxidative burst

**Fig 10:**
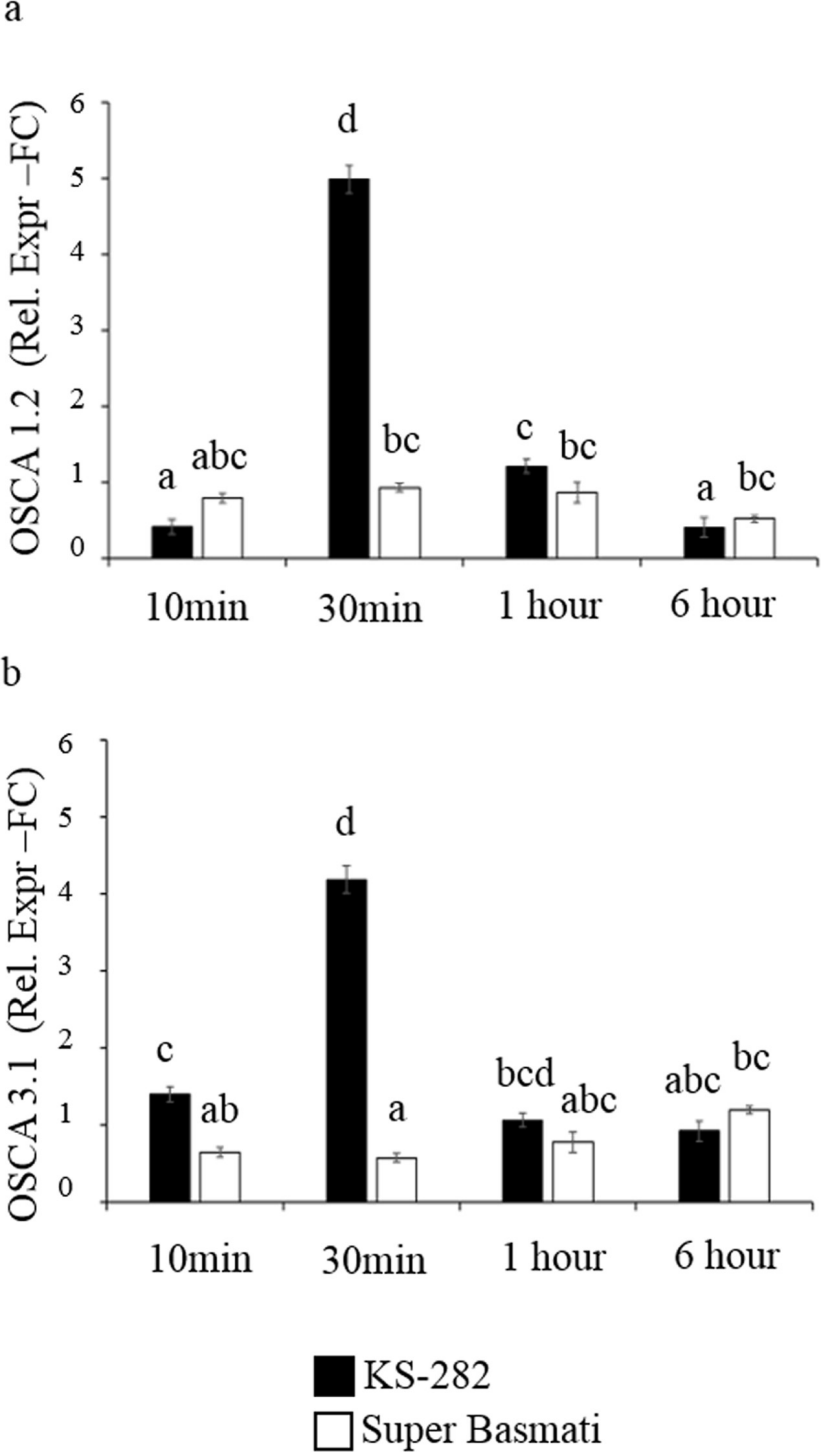
Effect of NaCl on the expression genes crucial for the osmosensing and Ca^2+^ influx. Relative expression (Fold Change) of (a) *OsOSCA* 1.2 gene and (b) *OsOSCA* 3.1 gene at different time points after treatment with 100 mM NaCl. Values represent the mean ± confidence interval of three independent experiments in duplicate. Different letters indicate statistically different expression level according to ANOVA (p<0.05).

Ca^2+^ is a signal molecule that has been proven to regulate H_2_O_2_ production [35–36]. However, it has also been proposed that under stress intracellular Ca^2+^ waves travel from cell to cell via unknown mechano-sensors activated by the apoplastic ROS produced by the activity of NOXs [8]. We analysed the expression pattern of Ca^2+^ channels *OsOSCA 1*.*2* and *OsOSCA* 3.1 up to 6 h of salt treatment. We were able to see a significantly high expression of *OsOSCA 1*.*2* and *OsOSCA3*.*1* at 30 min of salt treatment in tolerant KS-282 cultured cells (Fig 10A and 10B) which correlates positively to the burst of H_2_O_2_ in the cell cultures. No such expression of these two channels was seen in the control cell cultures of KS-282 (Supporting Information, S1A and S1B Fig). These results suggest that the *OsOSCA* genes can be a target of the H_2_O_2_ signalling pathway. The activation of different OSCAs genes can lead to the modulation of post-perception calcium signalling leading to the acclimation of the cells to the stress. The concomitant induction of the regulatory subunit gene *RbohA* also suggests that NOXs and OSCAs can be a part of the mechanism of transmission of the stimulus from outer to inner cell layers in whole plants, as suggested by [8]. No such response is activated in the sensitive Super Basmati cell cultures up to 6 h. This delay might be responsible for the lack of appropriate mechanisms to activate the signalling pathways necessary for adaptation to salt stress in sensitive cells cultures of Super Basmati.

### Comparative expression of the candidate genes involved in sodium ion sequestration, extrusion and potassium ion homeostasis

**Fig 11:**
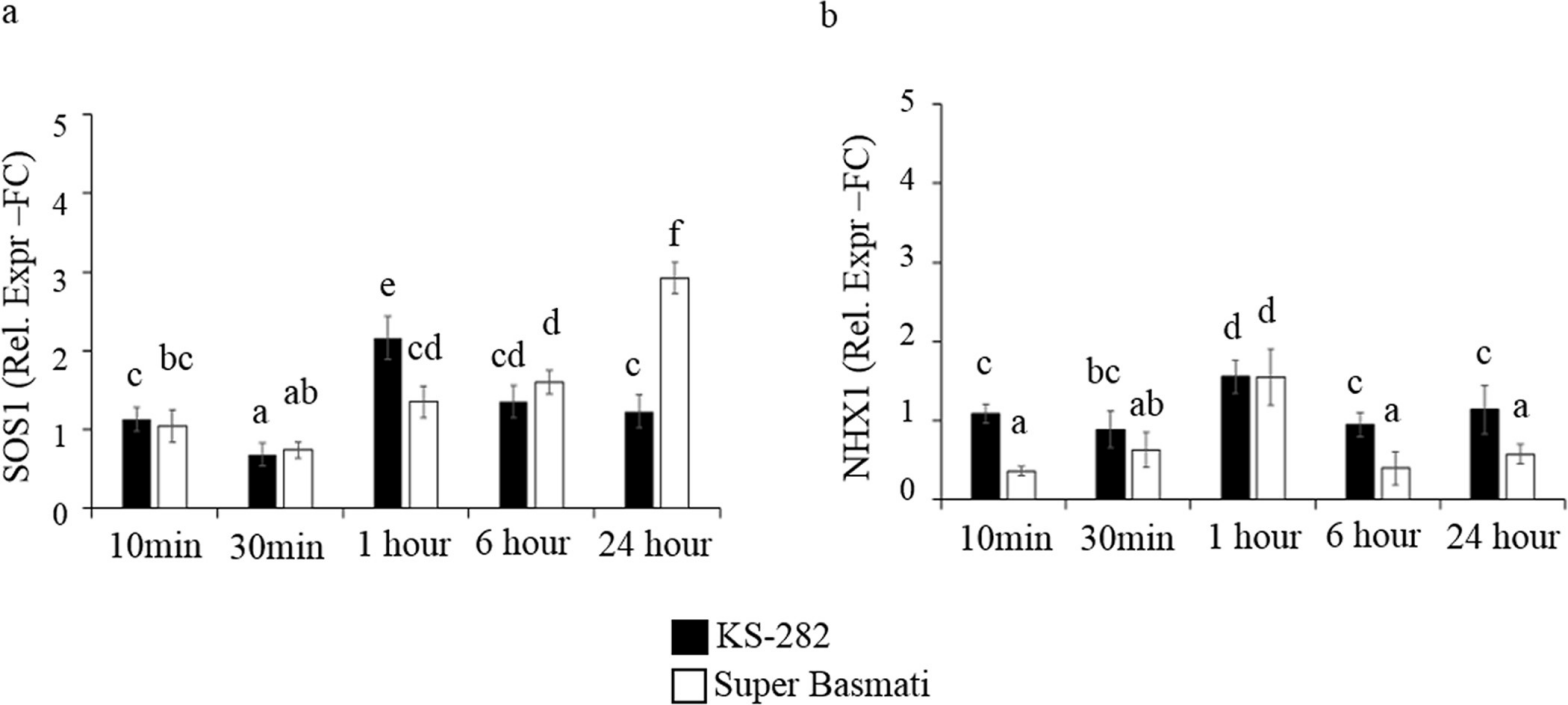
Effect of NaCl on the expression of genes crucial for the Na^+^ sequestration, extrusion. Relative expression (Fold Change) of (a) *OsSOS1* and (b) *OsNHX1* genes at different times after treatment with 100 mM NaCl. Values represent the mean ± confidence interval of three independent experiments in duplicate. Different letters indicate statistically different expression levels according to ANOVA (p<0.05).

To characterize the relationship between the oxidative burst, osmosensing Ca^2+^ channels and membrane ion transport proteins with respect to salt tolerance in indica rice, relative expression of *OsSOS1* (plasma membrane Na^+^/H^+^ antiporter; [37]) and *OsNHX1* (vacuole localized Na^+^/H^+^ antiporter; [38]) was analysed (Fig 11A and 11B). The difference in expression was analysed at time points starting from 10 min up to 24 h of salt treatment using RT-qPCR.

In the tolerant KS-282 cells, an up-regulation of *SOS1* was observed at 1h of salt stress generating a tolerance response at cellular level (Fig 11A) while in the sensitive Super Basmati cells a delayed expression was seen at 24 h. Similarly the expression of *NHX1* was comparatively and consistently higher in the tolerant KS-282 cells from 10 min to 24 h (except at 1 h) of salt treatment than the non-salt treated cell cultures (Supporting Information, S2 Fig) and sensitive Super Basmati cell cultures suggesting its important role in ion homeostasis.

**Fig 12:**
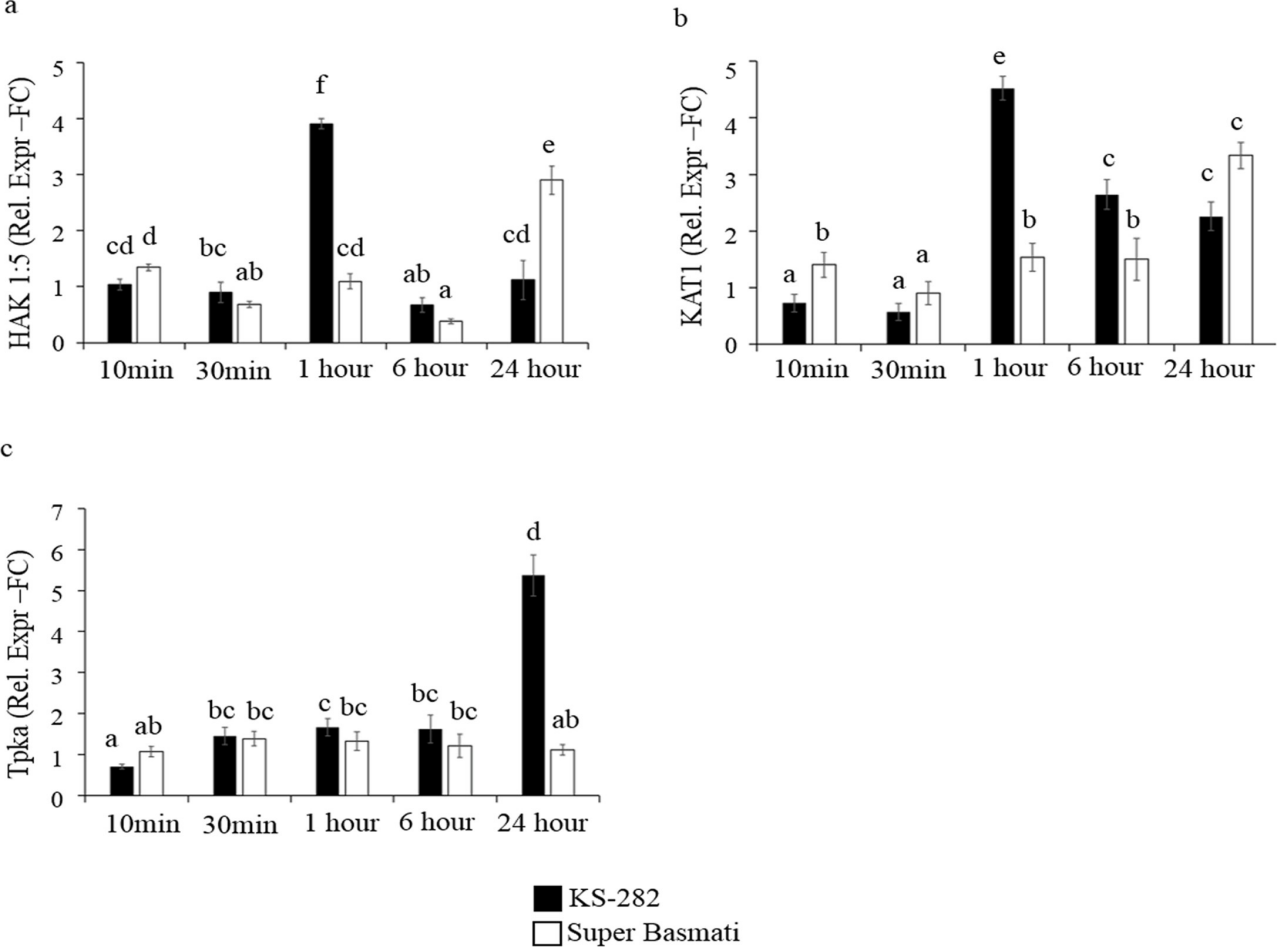
Effect of NaCl on the expression of genes important for K^+^ homeostasis. Relative expression (Fold Change) of (a) *HAK1*:*5*, (b) *OsKAT1* and (c) *OsTpka* genes at different times after treatment with 100 mM NaCl. Values represent the mean ± confidence interval of three independent experiments in duplicate. Different letters indicate statistically different expression levels according to ANOVA (p<0.05).

Maintaining a high [K^+^]/[Na^+^] ratio is an important trait of salt tolerant plants. Therefore, this trait was evaluated in KS-282 and Super Basmati cell cultures. Among K^+^ transporters analysed, the vacuolar localized two-pore K^+^ channels; *OsTpka* [39], involved in the release of K^+^ from the vacuole into the cytosol, showed a low expression in both the cell cultures in the first 6 h while its expression increased significantly only in the tolerant KS-282 cells at 24 h (Fig 12C). The plasma membrane located channel protein, high affinity K^+^ transporter *OsHAK5* [40] (involved in potassium uptake) and potassium channels genes important for salt tolerance, *KAT1* [41] was highly up-regulated in the tolerant variety at 1h of salt treatment showing an efficient K^+^ acquisition to maintain K^+^ homeostasis in the cytosol under saline condition (Fig 12A and 12B) as compared to the control group (Supporting Information, S2 Fig) and sensitive cell cultures of Super Basmati in which *OsHAK5* and *OsKAT1* expression was delayed till 24 h.

### Ion analysis

**Fig 13:**
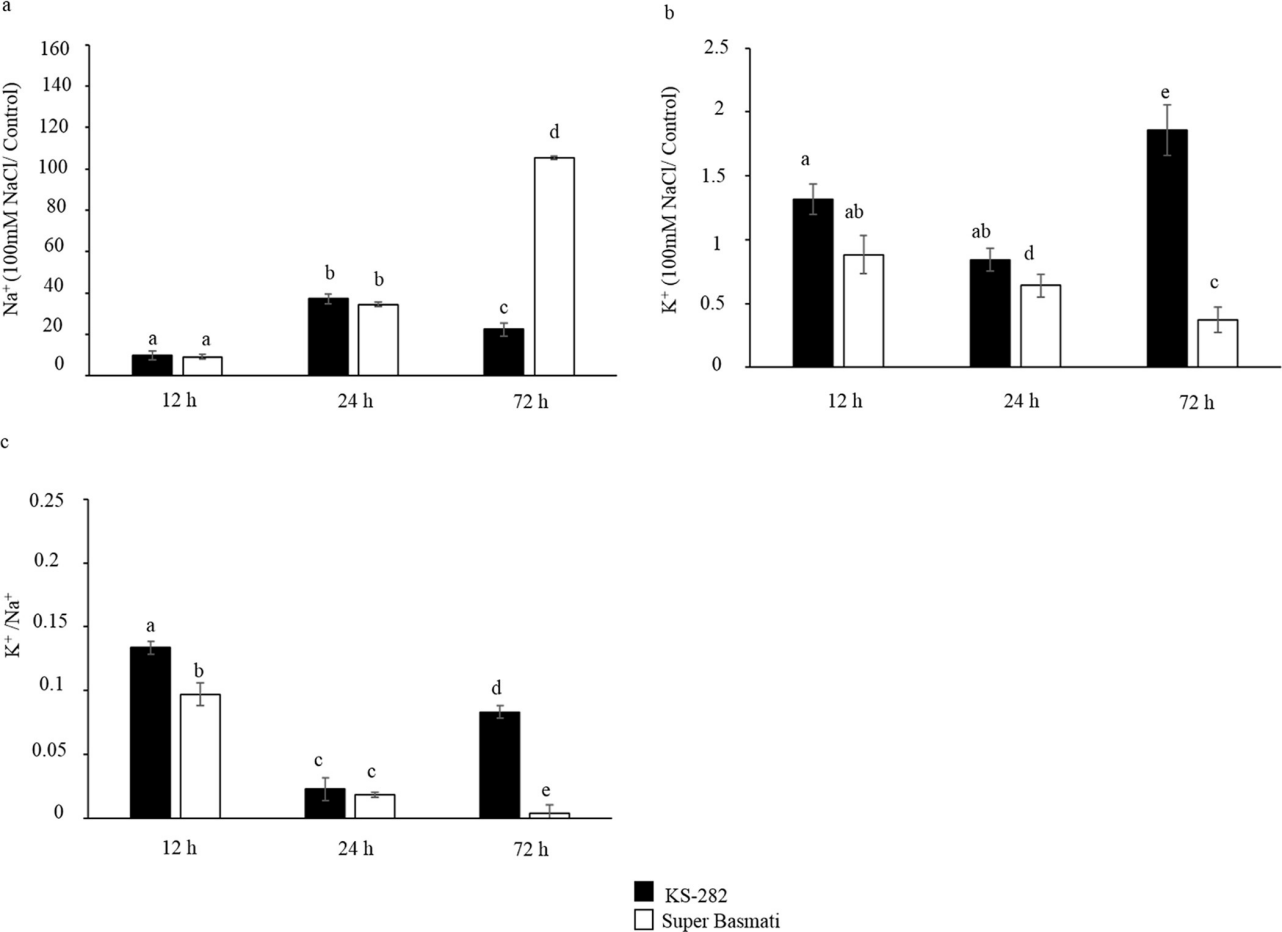
Effect of NaCl on the Na^+^ and K^+^ concentration (μg/g dry weight). Na^+^(a) and K^+^ (b) content (100 mM NaCl treated/ Control) in cells at different time points. The K^+^/Na^+^ ratio (c) as calculated represented a better K^+^ ion homeostasis in tolerant KS-282 cells as compared to the sensitive Super Basmati cells. Values represent the mean ± SE. Different letters indicate statistically different expression levels according to two-way ANOVA (p<0.05).

Ion analysis revealed a decrease in K^+^ content in both cell cultures at 24 h of salt treatment however cells of tolerant KS-282 were able to increase K^+^ concentration with a decrease in Na^+^ content at 72 h of salt exposure. On the other hand, in case of the sensitive Super Basmati cells, a sharp increase in the Na^+^ content with a progressive decrease in K^+^ concentration was observed (Fig 13A and 13B). Similarly, the tolerant cell cultures were able to maintain a higher K^+^/Na^+^ ratio at 12 and 72 h after salt treatment whereas in the sensitive cells a sharp decrease was recorded.

### Differential expression of genes upregulated by salt stress or Abscisic acid

In order to define whether the differences in expression profiles of ROS- and ion homeostasis-related genes were related to difference in perception of the salt stress, we analysed the expression profile of genes known to be up-regulated by salt or ABA i.e. *OsLEA19*,*OsRAB16*, *PYL4 and ABA45*. LEA and dehydrins are known to accumulate upon salt and osmotic stress. *OsLEA19* and *OsRab16*, in particular, have been demonstrated to confer salt tolerance in rice [42,43]. *ABA45* harbours the GRAM motif typical of genes in the ABA signalling pathway and it is known to be upregulated by ABA administration [44]. *PYL4* belongs to the family of Pyrabactins-like receptors for ABA [44].

**Fig 14:**
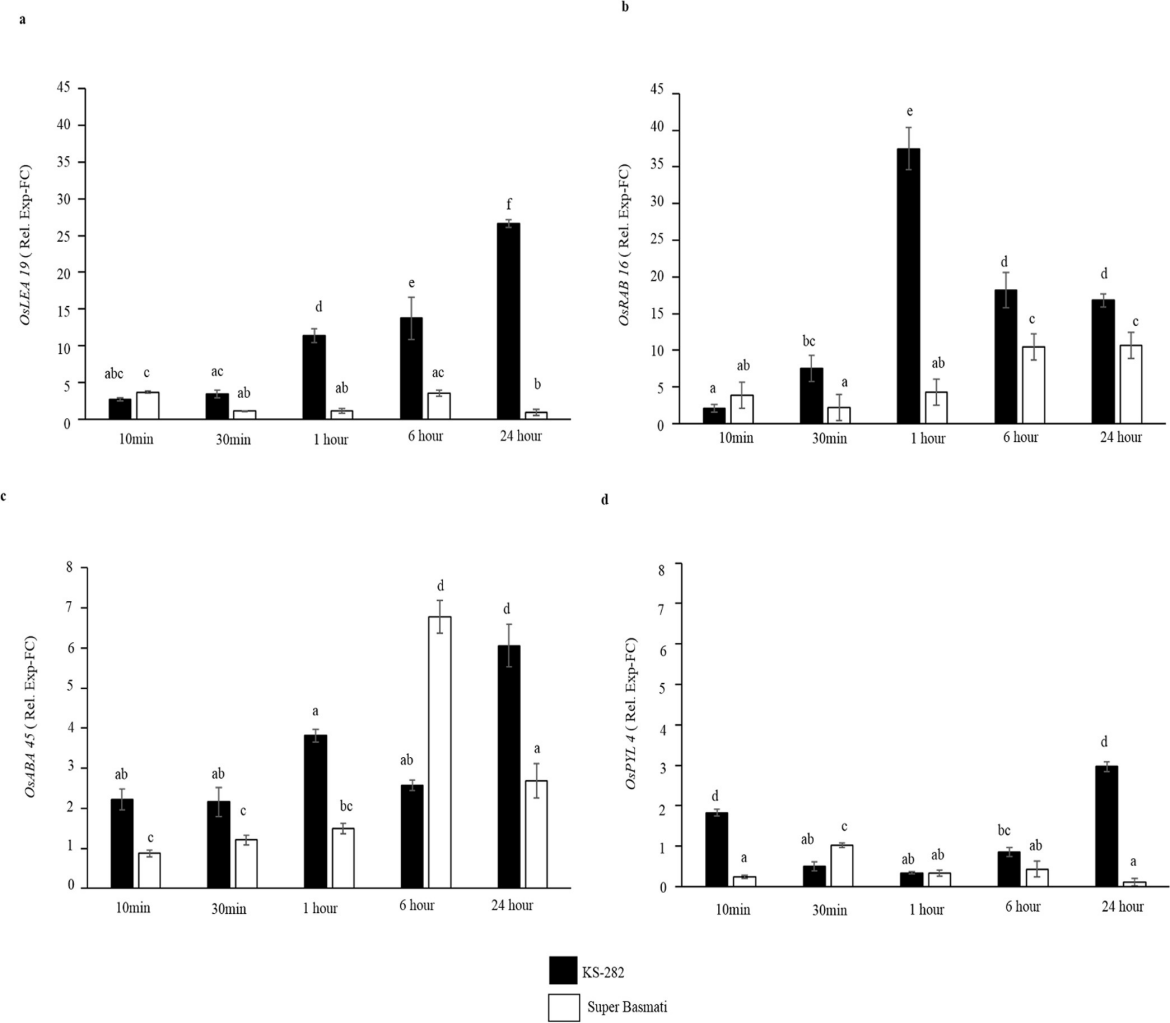
Effect of NaCl on the expression of genes involved in salt stress and ABA signalling. Relative expression (Fold Change) of (a) *OsLEA19*, (b) *OsRAB16* (c) *OsABA45* and (d) *OsPYL4* genes at different times after treatment with 100 mM NaCl. A stronger and higher expression of theses gene suggest a strong correlation between their expression and differences in salt stress perception between the two cell cultures. Values represent the mean ± confidence interval of three independent experiments in duplicate. Different letters indicate statistically different expression levels according to ANOVA (p<0.05).

All these genes showed differences in the expression level between the two varieties under salt conditions (100mM NaCl, Fig 14A, 14B, 14C and 14D). An early (10–30 min) and stronger response of *OsLEA19*, *OsRAB16* and *OsABA4*5 was measured in tolerant cells. Our data strongly suggests that very early events in the tolerant system were put in place that led to the regulation of salt responsive genes and eventually the survival of the cells.

## Discussion

ROS produced by the apoplastic NADPH oxidase plays a crucial role in mediating stress tolerance during the initial phase of salt stress. These ROS might act as an acclimation signal activating H_2_O_2_ dependent molecular signaling cascade. Here, we propose a salt stress induced signaling pathway in the tolerant cell cultures, positively regulated by calcium influx modulation via mechano-sensitive OSCA channels and activating ROS dependent TFs *OsSERF1*and *OsDREB2A*, which propagates the initial transcriptional response under salt stress in rice.

We generated cell cultures from the mature seeds of two indica rice varieties which have previously shown contrasting response to salt stress. We demonstrated that, on treatment with 100mM NaCl, KS-282 cells are able to set up specific tolerance mechanisms, whereas Super Basmati cells undergo cell death. Growth parameters along with measurements of cell death induced by treatment was determined by comparing the growth curves and cell death percentage (CDP) revealing differences in salt sensitivity. The cell cultures of sensitive Super Basmati showed a marked reduction in the fresh weight (FW) under salt stress when compared to the lines grown in standard conditions. By measuring the CDP at different time points during sub-culture cycles, cultured cells from Super Basmati reached a higher CDP after 6 days of salt treatment at 150 and 100 mM NaCl respectively (Fig 2B) as compared to the tolerant KS-282 cells validating these cell cultures to be highly tolerant to salt stress and making them suitable for studying the stress response.

Previous data indicate that under salinity stress, reactive oxygen species, notably H_2_O_2_ interact with NO [45]. The possibility that NO works together with the universal second messenger Ca^2+^ in plant signaling processes has also been proposed [46,47]. To understand the cross talk between these important signaling components we compared the extra and intracellular H_2_O_2_ and intracellular NO levels in the two cell cultures. The two cell cultures differed with respect to extracellular and intracellular H_2_O_2_ and NO accumulation upon salt treatment. In the sensitive variety, salt stress induced a delayed but high level of extracellular H_2_O_2_ at 24 h (Fig 3C) after salt stress while intracellularly H_2_O_2_ was generated within the first 5 min of treatment which reached at a maximum amount at 3 h (Fig 4D) together with a progressive NO increase over time (Fig 7B). The level of H_2_O_2_ remained high up to 48 h intracellularly in a dose dependent manner which is consistent with the activity of SOD as measured under 100mM NaCl. On the other hand, in tolerant KS-282 cells exposed to salt stress, an initial extracellular H_2_O_2_ signal was detected at 15 min (Fig 3A). While a later burst of H_2_O_2_ (possibly responsible for intercellular ROS wave) was seen at 24 h. Previously, it has been demonstrated in rice that NaCl stress triggers H_2_O_2_ production in roots within 5 min, and this increase depends on NADPH oxidase activity [24]. Intracellularly, at 5 min and 3 h H_2_O_2_ was detected which optimised to basal level by the high antioxidant enzyme activity (tAPX/ cAPX and CAT) indicating a control over the rapid oxidative burst (Figs 5A, 5B, 6A and 6B). An increase in the NO level was observed at 1 h (Fig 7A) which declined after 3 h and reached to basal levels bringing the cell cultures to a controlled oxidative state and precluding cell death in the tolerant cells.

The initial high level of extracellular H_2_O_2_ in the tolerant KS-282 were found to be consistent with the expression of H_2_O_2_- responsive and salt-specific TF *SERF1* and downstream genes *DREB2a* and *OsRbohA* genes as revealed by expression analysis. This early oxidative burst has been previously associated with stress tolerance in plants [48] suggesting H_2_O_2_ as a possible signalling molecule. This prompt increase in *OsRbohA* transcript is consistent with the finding that *OsRbohA*-overexpressing transgenic lines exhibit much greater drought tolerance [49] and *A*. *thaliana* mutants lacking *RbohF* (*OsRboh*A gene homologue) showed decreased salinity tolerance [50]. Notably, in KS-282 cells, the expression level of *SERF1* and *DREB2a* (Figs 9A and 6B), two genes involved in salt-induced signalling processes [25,24], increased very rapidly and earlier after salt stress exposure than in Super Basmati cells. Consistent with our observation, *SERF1* has been reported to act as a positive regulator of salt tolerance [25]. Along with the extracellular H_2_O_2_ signal, a sharp increase in NO level was seen in tolerant KS-282 cell cultures which dropped to basal level after 3h of salt treatment while in the case of sensitive cell cultures, huge amounts of NO were generated within the first 1h which then reduced but remained above basal levels up to 72 h of salt treatment indicating its continuous production. RBOH at the plasma membrane are known to be regulated by NO [8] through S-nitrosylation suggesting interplay between NO signalling and ROS homeostasis [51] which is maintained by a higher APX/CAT activity and a low SOD activity in the tolerant cell cultures. NO plays a dual role during systemic signaling by amplifying or dampening the signal [52,53]. In case of the sensitive Super Basmati cells, no extracellular H_2_O_2_ and *RbohA* expression was seen up to 24 h of salt stress while a high level of intracellular H_2_O_2_ was observed along with a significantly high SOD activity and a low APX and CAT activity. Consistently high level of intracellular NO in the sensitive cell cultures after salt exposure suggest its role in inhibiting ROS scavenging enzymes reducing H_2_O_2_ decay inside the cells causing more oxidative damage and consequent programmed cell death. NO mediated S-nitrosylation inhibiting antioxidant enzymes i.e. catalase and ascorbate peroxidase has already been proposed by several groups [54,55] and our results are consistent with previous data. NO radical when scavenged by cPTIO prevents this damage, reducing the cell death rate as evident by a significantly decreased CDP in both sensitive and tolerant cell cultures. While in the case of tolerant KS-282 cells, a single peak of NO up-regulates the NOX activity and H_2_O_2_ signal within 3h of salt stress. In Arabidopsis, *AtRbohD* was shown to be upregulated through a NO-dependent process elicited by oligo-galacturonides in response to pathogen attack [52]. Improved salt tolerance has been reported in *Arabidopsis thaliana* with enhanced GST activity and a contolled ROS signal [56, 57]. Under hypoxia and salt stress, NADPH oxidase (s) (*RbohD*) play a major role in producing and controlling ROS signal [34] via K^+^ homeostais and reduced Na^+^ accumulation by pumping Na^+^ out of the cytosol [58]. Moreover, NO accumulation has been correlated with reduced growth rate under low oxygen stress predicting a cross talk between ROS and NO signal [59]. Similarly, the findings of these studies augment the observed link between NO and reduced growth rate of cell cultures in our study under salt stress.

It is well established that various biotic and abiotic stimuli trigger an increase in the intracellular Ca^2+^ levels by the activation of unknown Ca^2+^ channel [21,60–63]. Calcium acts as an important second messenger in the signal transduction of many abiotic stress responses [64]. *OSCAs* channels, activated by hyperosmolality, have been shown to be involved in osmotic-stress induced fast signaling events [65], suggesting *OSCAs* to be osmosensor. These mechanosensitive Ca^2+^ channels, *ATOSCA 1*.*1* and *ATOSCA 1*.*3* have recently been characterized in Arabidopsis and reported to play an important role in osmosensing under salt stress [66]. The role of Ca^2+^ influx in affecting *ATRboh* activity is instrumental and associated with ABA signaling, Ca^2+^ modulation and eventually is involved in salt stress adaptation [67, 58]. In silico characterized members of OSCA family i.e. *OSCA 1*.*2* and *OSCA3*.*1* genes in rice were analyzed in present study. Consistent with the expression of genes involved in oxidative burst, the expression of *OSCA 1*.*2* and *OSCA3*.*1* channel proteins was significantly higher in tolerant KS-282 cell cultures in the first 30 min of salt treatment while a weaker response of these channel was observed in the sensitive cell culture. Previously a mechanism involving an increase in intracellular Ca^2+^ due to apoplastic oxidative burst has been proposed [8]. ROS-activated Ca^2+^ channels and transporters in the plasma membrane have been identified at the electrophysiological [68–70] and molecular levels [42]. We propose that *OSCA 1*.*2* and *OSCA3*.*1* channels are modulated by H_2_O_2_ and may be related to systemic signaling by increasing an influx of Ca^2+^. Our results regarding ROS wave and consequent Ca^2+^ influx are in agreement to the recent study available in japonica rice plants suggesting a particular composition of OSCA and RBOH at the plasma membrane capable of responding to salinity stress and adaptation [43]. We propose that once such an initial ‘priming’ Ca^2+^ influx has occurred, the resulting cytosolic Ca^2+^ signal is amplified through vacuolar stores triggering long range signaling in which specific genes are involved (*OSCA 1*.*2*, *OSCA 1*.*3* and *RbohA*).

Maintaining low cytosolic Na^+^ levels while keeping high levels of K^+^ inside the cells is well-reported as an effective strategy to cope with salt stress. The maintenance of a high cytosolic [K^+^]/[Na^+^] ratio is crucial for salt tolerance [44]. The expression analysis of genes involved in increasing cytosolic K^+^ concentration correlated with our ion analysis data and showed that KS-282 cells upregulate *HAK5* and *KAT 1* earlier than Super Basmati cells. The expression of *OsTPK1a* in the tolerant KS-282 was seen after 24 h while its expression remained low in the sensitive cell culture. The significantly high expression of some K^+^ transporter/channel genes has been reported to increase salt tolerance [40,71–73]. Therefore, in salt-tolerant KS-282 cells, a response to salt stress seems to involve the capability to more efficiently maintain K^+^ homeostasis. Our results are further supported by the expression analysis of genes (*OsLEA19*; *OsRAB16; ABA45*) that have been demonstrated to be induced upon salt, osmotic stresses and ABA [74–76]. Moreover, after a massive increase in Na^+^ content in both varieties, only KS-282 cell cultures were able to reduce intracellular Na^+^ levels within 72 h of salt treatment. Therefore, we can hypothesize that the higher intracellular H_2_O_2_ level observed in treated Super Basmati cells undergoing programmed cell death can also be correlated with the reduced [K^+^]/[Na^+^] ratio. No significant differences in the expression of *NHX1* in the two cell cultures also suggest that in KS-282 cell cultures, tolerance may depend more on increased cytosolic[K^+^] to sustain a high [K^+^]/[Na^+^] ratio than on Na^+^ compartmentalization, while Na^+^ exclusion does not play an important role during the early response to salt stress.

## Conclusion

In essence, we demonstrate that salt tolerance in KS-282 cells depends mainly on an efficient stress perception mediating a control over ROS homeostasis via upregulated ROS scavenging enzyme activities and its ability to maintain a high [K^+^]/[Na^+^] ratio. The ability of KS-282 cells to generate an apoplastic H_2_O_2_ burst and to maintain low intracellular levels of H_2_O_2_ plays a key role in survival. The H_2_O_2_ signal, generated by apoplastic NOX and synchronised with the NO signal along with a high influx of Ca^2+^ (putatively through OSCA hyperosmolality-gated channels), causes an upregulation of *OsSERF1* and *OsDREB2A* TFs and generates a vital response in tolerant cells. Conversely, the sensitive cell culture fails to produce a timely ROS and Ca^2+^ signal. Intracellularly, lethal amounts of H_2_O_2_ and NO trigger programmed cell death. Along with that the tolerant cell cultures have the ability to maintain a high K^+^ concentration due to a rapid increase in the expression of genes coding for transporters/channels localized to the tonoplast and to the plasma membrane.

## Supporting information

S1 Table List of primers used in this study. (DOCX)

S1 Fig The expression patterns of genes involved in early Ca^2+^ and ROS signal before NaCl treatment. In the control cell cultures of KS-282 and Super Basmati the expression of OSCA channels 3.1 and 2.1 (A and B) was low and similarly, The expression of genes encoding trascription factors involved in ROS signalling was silent in cell cultures at diffrent time points before salt treatment.
(TIF)

S2 Fig The expression patterns of genes involved in Na^+^ homeostasis before NaCl treatment. As measured in the non salt treated cell cultures of KS-282 and Super Basmati the expression of channels and transporters involved in Na^+^ homeostasis was consistantly low in both cell cultures at diffrent time points.
(TIF)

S3 Fig Antioxidant enzyme, Dehydroascorbate reductase and mono-dehydroascorbate reductase activities in control and treated cells. The activity of Dehydroascorbate reductase (A and B) and mono-dehydroascorbate reductase (C and D) was measured at 30 minutes and 24 hours of salt treatment in the salt tolerant and sensitive cell cultures. No difference was observed in the antioxidant activity predicting its secondary role in redox homeostasis.
(TIF)

S4 Fig Antioxidant enzyme glutathione reductase activities in control and treated cells. The activity of glutathione reductase was measured at 30 minutes and 24 hours of salt treatment in the salt tolerant and sensitive cell cultures. No difference was observed in the antioxidant activity predicting its secondary role in redox homeostasis.
(TIF)

S1 File Underlying data. (DOCX)
