# Supplementary material for: Salt tolerance in indica rice cell cultures depends on a fine tuning of ROS signalling and homeostasis
Source: PLoS One. 2019 Apr 30;14(4):e0213986. doi: 10.1371/journal.pone.0213986 (PMC6490951; doi:10.1371/journal.pone.0213986)
Supplement: S1 Table — (DOCX) [file pone.0213986.s001.docx]

**S1 Table. List of primers used in this study**

| **Name** | **RAP_ID** | **forward** | **reverse** |
| --- | --- | --- | --- |
| ubiquitin | Os05g0160200 | TTCTACAAGGTGGACGACGC | AGATCAGAGCAAAGCGAGCA |
| KAT1 | Os01g0756700 | TTTCGACGATGGCGACAGAT | GGTCATAAGGCGAGACGAGG |
| TPK1a | Os03g0752300 | GAGATCTACACCGAGCGACG | GAGTGGTCGACATCGAGCTT |
| HKT1;5 | Os01g0307500 | TCGTCATCTGCATCACGGAG | CATGACGGCCATGAGAGTGA |
| HAK5 | OS01g0930400 | ACACTGAGAATGACCGAGCC | TTCCAGCTTCCATGTGCAGT |
| NHX1 | Os07g0666900 | ACCGTGAGGTTGCCCTTATG | CAGAGTTGCAAATGCGTGCT |
| SOS1 | Os12g0641100 | TGAGATAGGGAGGCCCGAAA | TCATGCTCCCGTACATGCTC |
| SERF1 | Os05g0420300 | GAGTGAGGAGCTCATTGTTTACGA | ACATCAAAATTTCCATGTCATCTA |
| RBOHA | Os01g0734200 | TGGCTAAGGCCAGAAGCAC | GCCCAACCTGGAGCCAATAA |
| DREB2A | Os01g0165000 | GGCTGAGATCCGTGAACCAA | AATTGACACGTGCTGTGGGA |
| LEA19 | Os05g0542500 | CGGCAGCGTCCTCCAAC | CGGTCATCCCCAGCGTG |
| Rab16 | Os11g0454300 | ACACCACAGCAAGAGCTAAGTG | TGGTGCTCC ATCCTGCTTAAG |
| ABA45 | Os01g0702500 | AGAGAGGGGACAGCCCGATG | AGGCTCAGCTTCCCCATCGC |
| PYL4 | Os03g0297600 | AGCCCCAGGCGTACAAGC | GTGGTGACCGAGAGGTAGTTC |
| OSCA1.2 | Os03g0397400 | GACGCCACGCACCAATTCTC | TCGGATCGATGGAGCCTGGA |
| OSCA3.1 | Os07g0150100 | TTCCCAGCTACGAGAGCAAC | ATTGGGATGAGGGGCACAAG |
