# Supplementary figures and images for: Salt tolerance in indica rice cell cultures depends on a fine tuning of ROS signalling and homeostasis
Source: PLoS One. 2019 Apr 30;14(4):e0213986. doi: 10.1371/journal.pone.0213986 (PMC6490951; doi:10.1371/journal.pone.0213986)

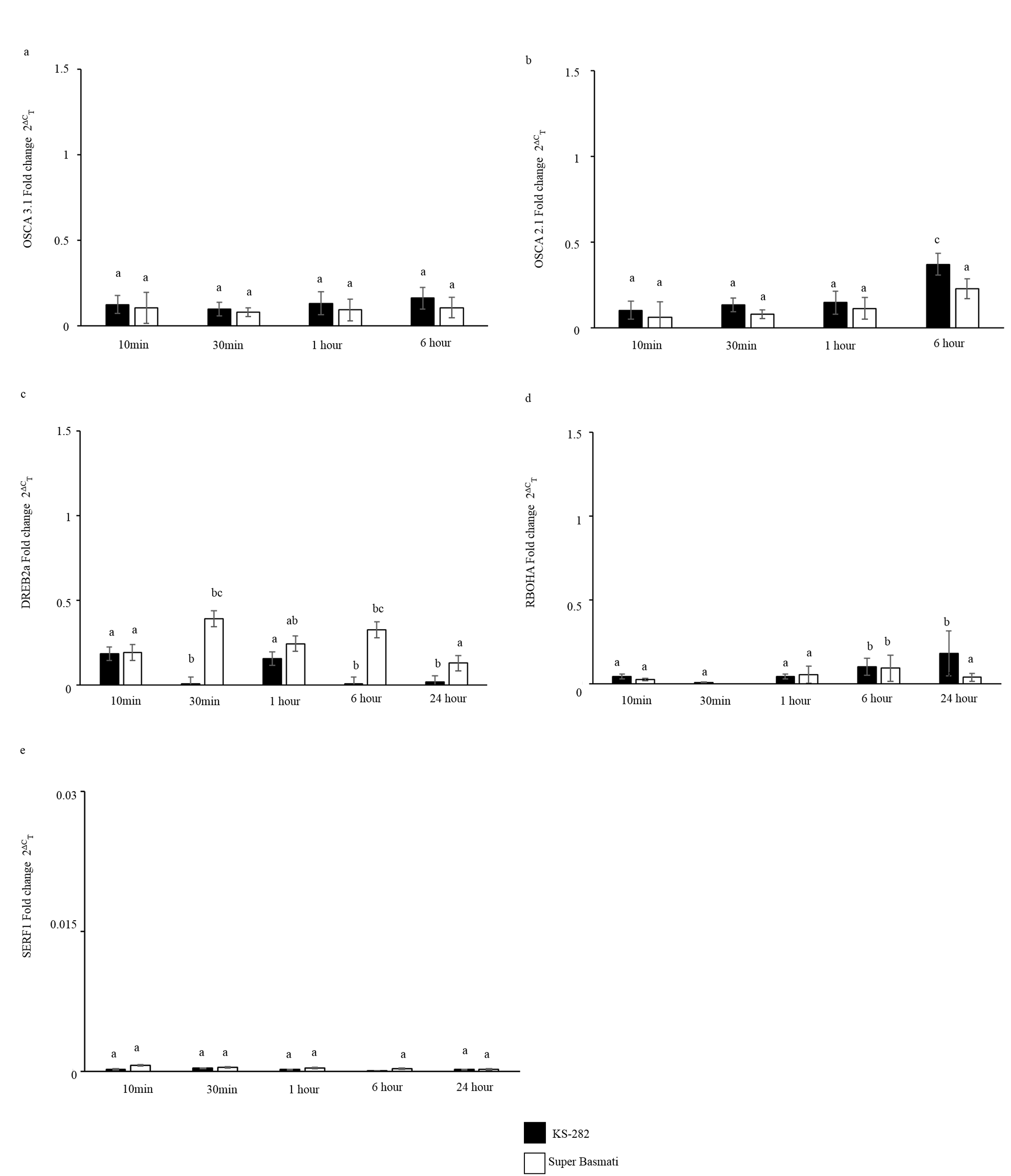

Supplement: S1 Fig — In the control cell cultures of KS-282 and Super Basmati the expression of OSCA channels 3.1 and 2.1 (A and B) was low and similarly, The expression of genes encoding trascription factors involved in ROS signalling was silent in cell cultures at diffrent time points before salt treatment. (TIF) [file pone.0213986.s002.tif]

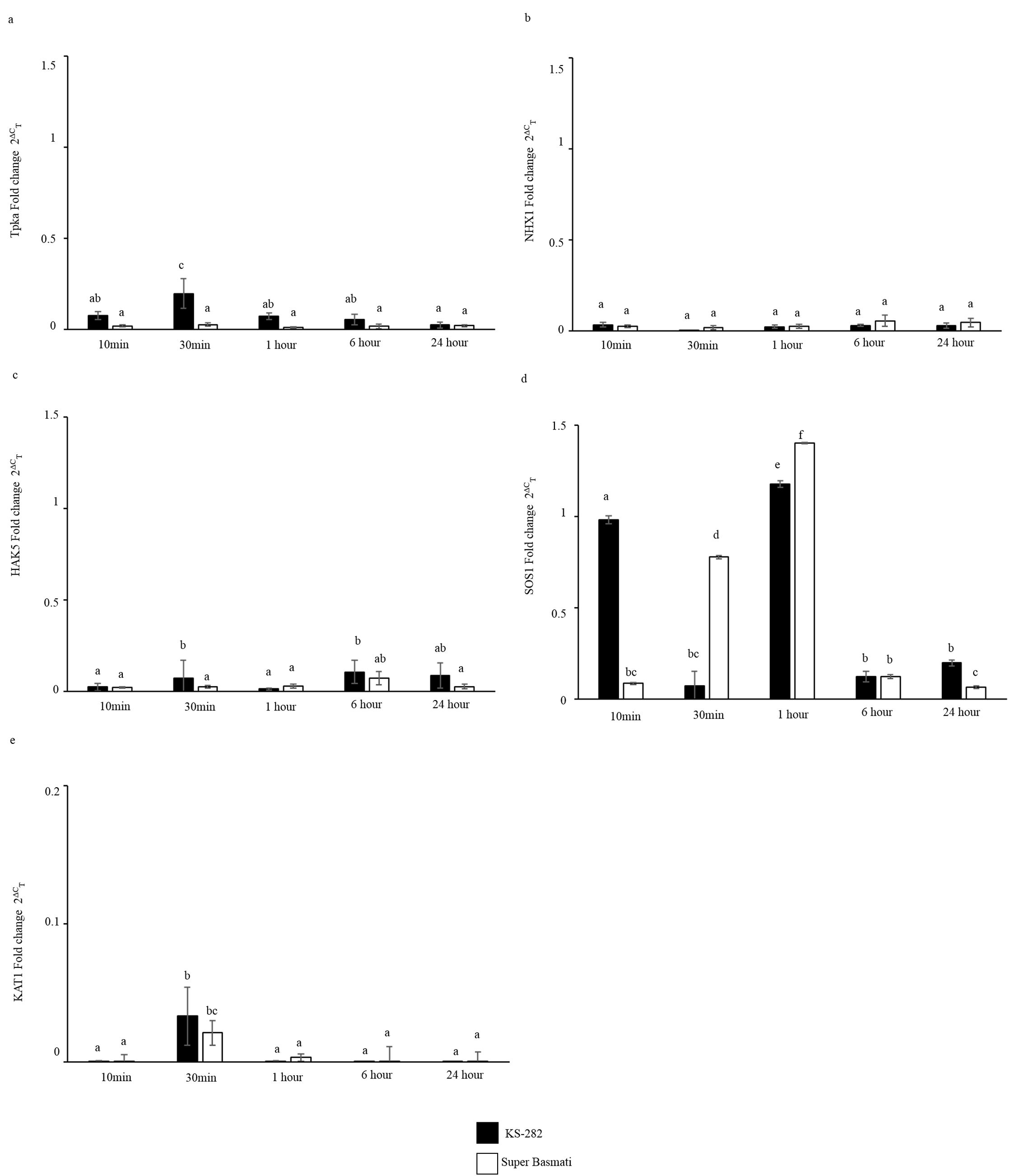

Supplement: S2 Fig — As measured in the non salt treated cell cultures of KS-282 and Super Basmati the expression of channels and transporters involved in Na+ homeostasis was consistantly low in both cell cultures at diffrent time points. (TIF) [file pone.0213986.s003.tif]

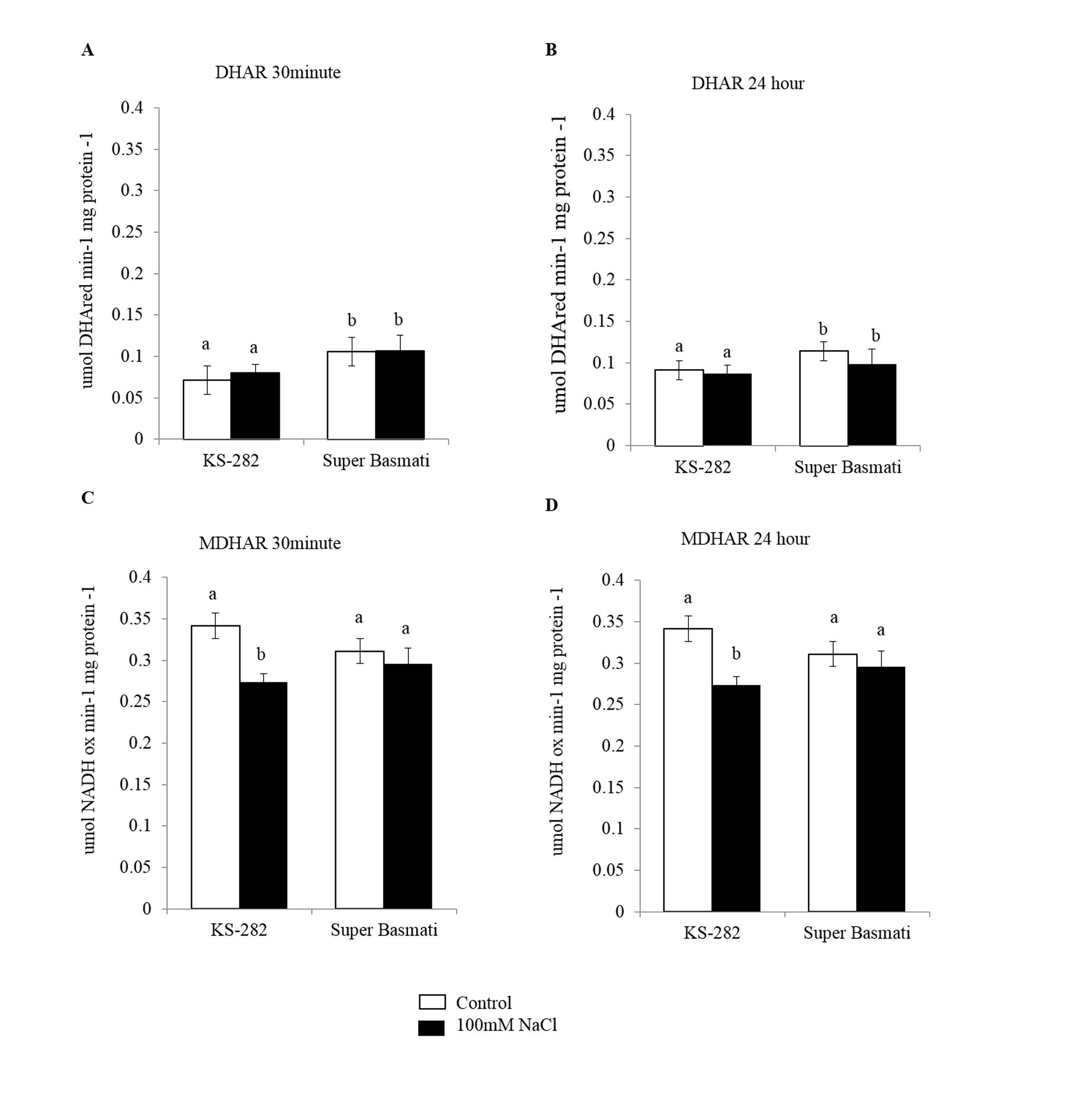

Supplement: S3 Fig — The activity of Dehydroascorbate reductase (A and B) and mono-dehydroascorbate reductase (C and D) was measured at 30 minutes and 24 hours of salt treatment in the salt tolerant and sensitive cell cultures. No difference was observed in the antioxidant activity predicting its secondary role in redox homeostasis. (TIF) [file pone.0213986.s004.tif]

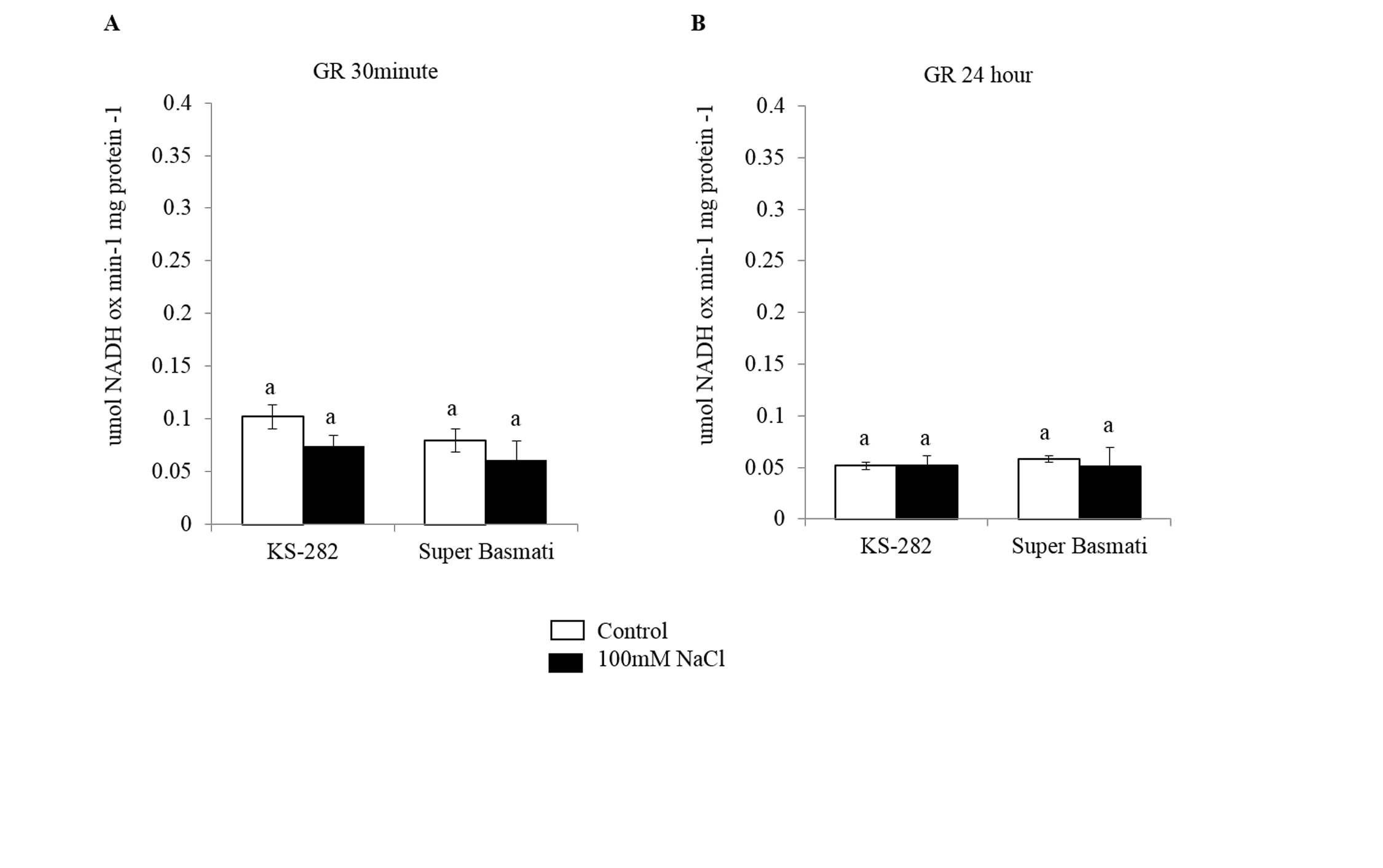

Supplement: S4 Fig — The activity of glutathione reductase was measured at 30 minutes and 24 hours of salt treatment in the salt tolerant and sensitive cell cultures. No difference was observed in the antioxidant activity predicting its secondary role in redox homeostasis. (TIF) [file pone.0213986.s005.tif]
