# Supplementary material for: Salt tolerance in indica rice cell cultures depends on a fine tuning of ROS signalling and homeostasis
Source: PLoS One. 2019 Apr 30;14(4):e0213986. doi: 10.1371/journal.pone.0213986 (PMC6490951; doi:10.1371/journal.pone.0213986)

**Supporting Information**

**Statistical analysis details**

**(TWO WAY ANOVA for Fig 1,2,3,4,5,7,8 and 13).**

**Fig 1.**

**Fresh Weight**

**Super Basmati**


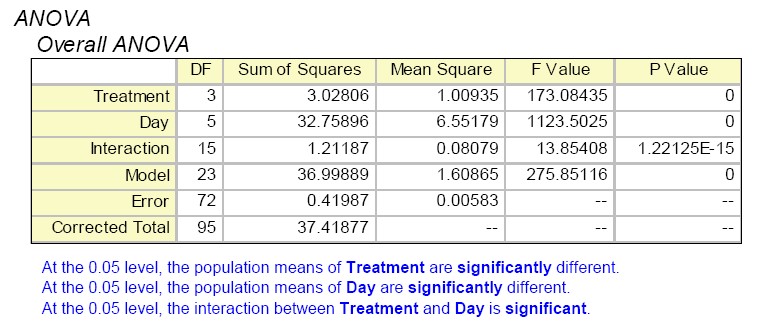
**Fresh Weight**

**KS-282**


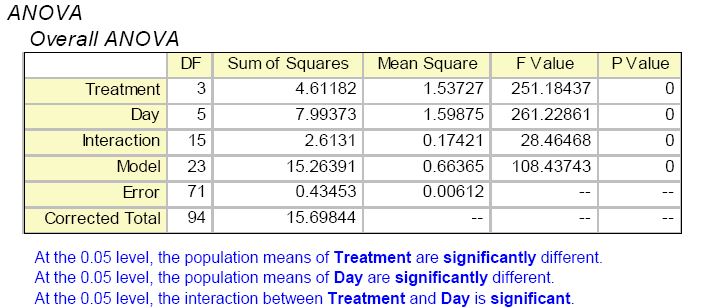


**Dry weight-**

**KS-282**


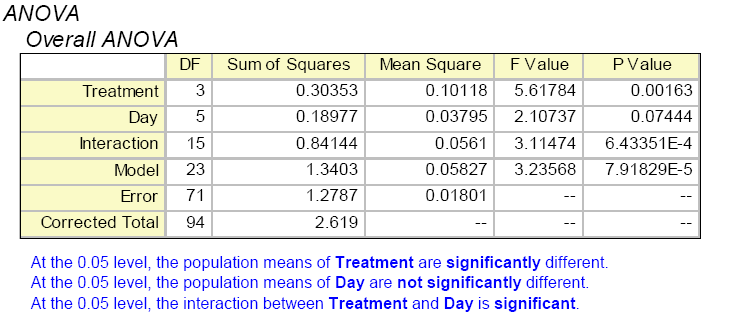


**Dry weight-**

**Super Basmati**


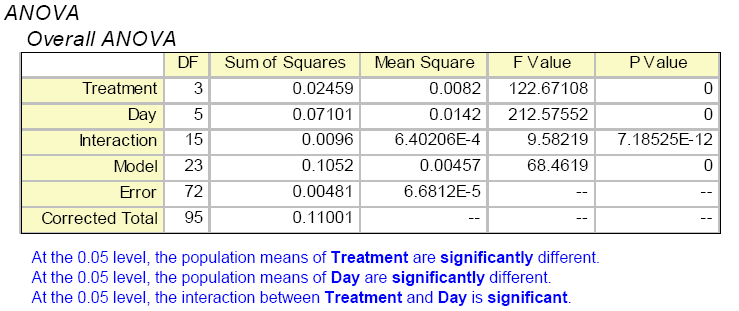


**Fig 2 Viability assay**

**Cell Death %**

**Super Basmati**


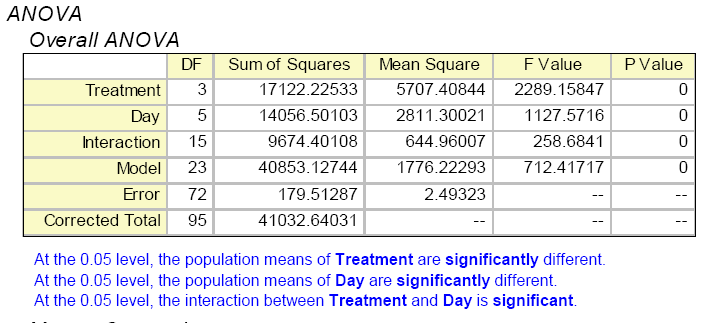


**Cell death %**

**KS-282**


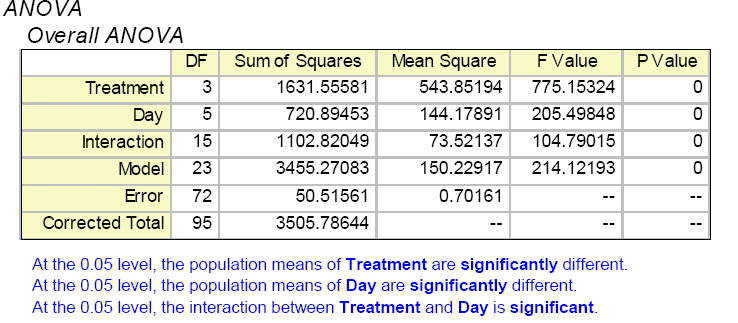


**Fig 3. Extracellular H_2_O_2_ produced by rice cell cultures**

**Extracellular H_2_O_2_**

**Super Basmati**


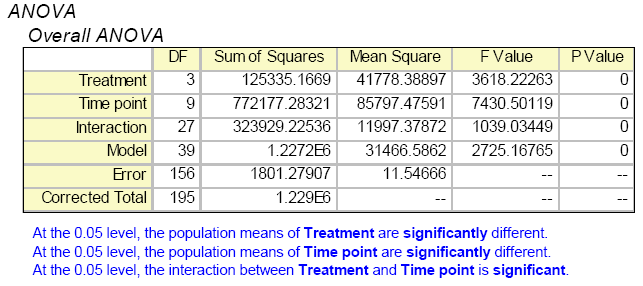


**Extracellular H_2_O_2_**

**KS-282**


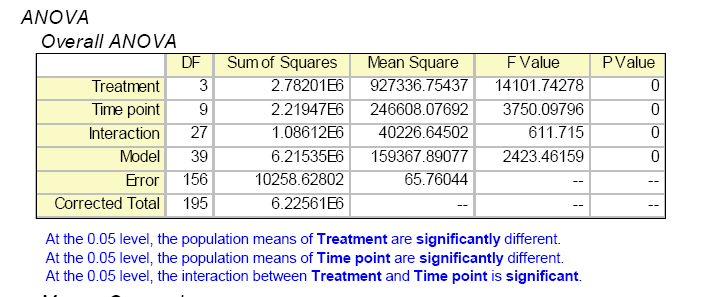


**Fig 4. Intracellular H_2_O_2_ produced by cell cultures**

**Intracellular H_2_O_2_**

**KS-282**


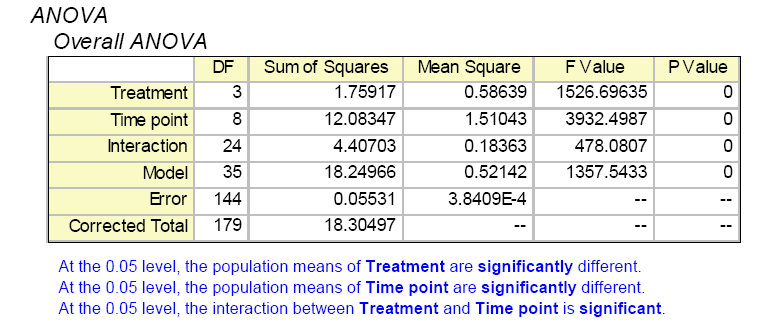


**Intracellular H_2_O_2_**

**Super Basmati**


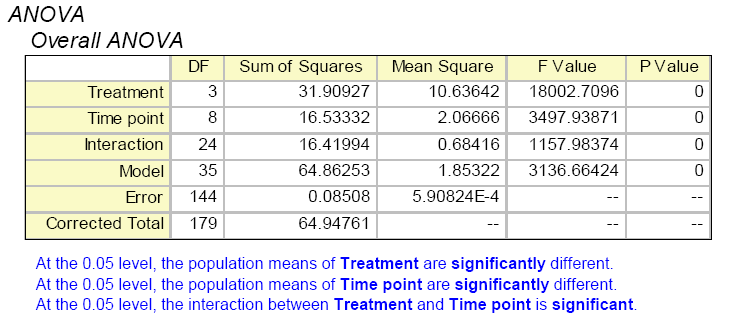


**Fig 7. NO produced by rice suspension cell cultures**

**NO**

**Super Basmati**


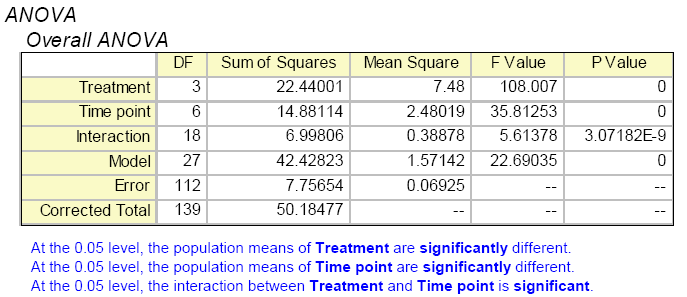


**NO**

**KS-282**


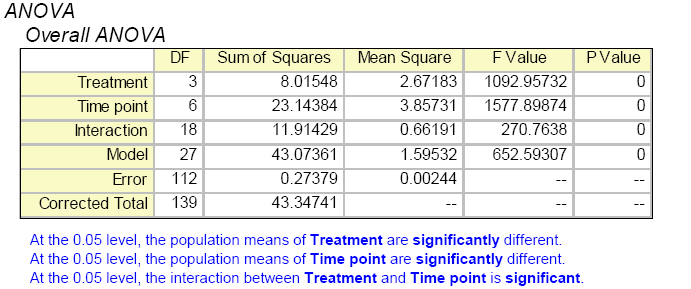


**Fig. 8. The effect of cPTIO pre-treatment on cell death percentage.**

**Viability assay with CPTIO**

**KS-282**


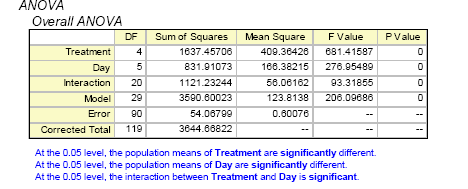


**Cell Death % with CPTIO**

**Super Basmati**


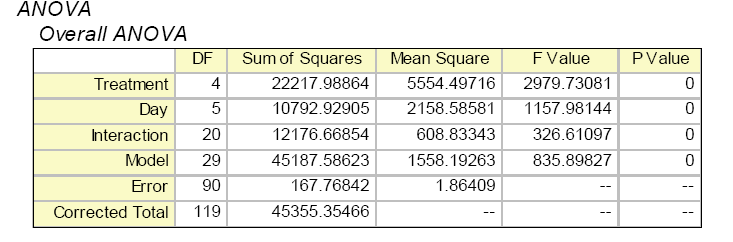


**Fig 13. Na^+^ and K^+^ content in the treated and control cell cultures.**

**Na^+^**

**Super Basmati**


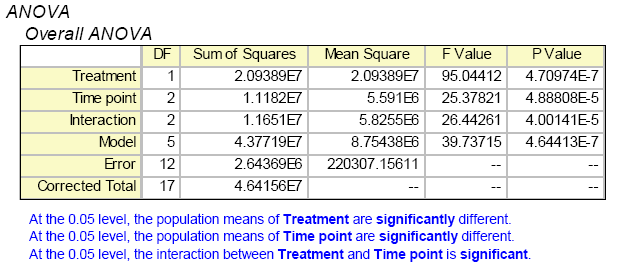


**Na^+^**

**KS-282**


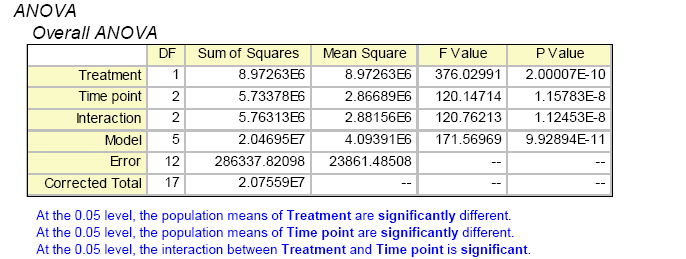


**K^+^**

**KS-282**


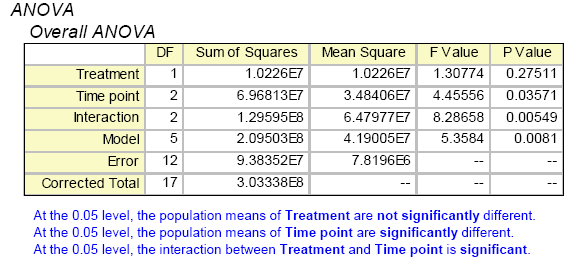


**K^+^**

**Super Basmati**


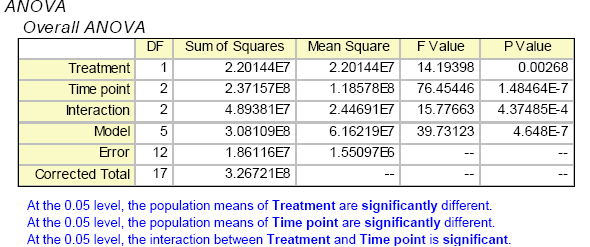

Supplement: S1 File — (DOCX) [file pone.0213986.s006.docx]
